# Downregulation of the m^6^A reader YTHDC2 upregulates exosome content in lung adenocarcinoma *via* inhibiting IFIT and OAS family members

**DOI:** 10.1016/j.jbc.2024.107783

**Published:** 2024-09-18

**Authors:** Zhixin Yin, Lifang Ma, Xiaoting Tian, Qi Sun, Congcong Zhang, Yikun Wang, Yayou Miao, Xiangfei Xue, Yongjie Wang, Jiayi Wang, Xiao Zhang, Xumin Hou

**Affiliations:** 1 Department of Clinical Laboratory Medicine, Shanghai Chest Hospital, Shanghai Jiao Tong University School of Medicine, Shanghai, China; 2 Shanghai Institute of Thoracic Oncology, Shanghai Chest Hospital, Shanghai Jiao Tong University School of Medicine, Shanghai, China; 3 Department of Cardiology, Shanghai Chest Hospital, Shanghai Jiao Tong University School of Medicine, Shanghai, China

**Keywords:** cancer, METTL3, RNA stability, RNA interaction, immunoprecipitation

## Abstract

N^6^-Methyladenosine (m^6^A) is the most prevalent mRNA modification. Its biological function primarily relies on its "Reader" protein, such as YTHDC2. Previous studies have shown that YTHDC2 downregulation is a procarcinogenic phenomenon in lung adenocarcinoma (LUAD). However, further investigation is needed to understand the molecular mechanisms of downstream genes and the associated biological phenomena following YTHDC2 downregulation. Here, we found that YTHDC2 knockout upregulated exosome content in LUAD. Following YTHDC2 knockout, the mRNA levels of OAS family members (OASs) and IFIT family members (IFITs) also decreased; and inhibition of OASs and IFITs could promote exosome content. Several m^6^A modification sites on the NT domain of OASs and the TPR12 domain of IFITs were found to increase the stability of OASs and IFITs in a YTHDC2-dependent manner. OASs and IFITs affected exosome content through target genes including RAB5A, RAB7, and RAB11A, and three arginine (R) amino acids on IFITs were critical for combination IFITs with targeted RAB mRNAs and subsequent degradation. Simultaneously, OASs degraded targeted RABs through RNAseL. Additionally, mutual bindings between OASs and IFITs were critical for their target gene degradation. Collectively, the above findings might provide a theoretical basis for the treatment of LUAD patients with low YTHDC2 expression.

Cancer is the leading cause of death worldwide, with 2.3 million new cases of lung cancer in 2020 (1). The number of new lung cancer cases in China is 870,982 in 2022 (2). By 2035, the incidence of lung cancer will continue to rise in most countries, and it will remain one of the biggest public health challenges in the world (3). As the most common pathological type of lung cancer, lung adenocarcinoma (LUAD) is one of the most malignant tumors, and the 5-years survival rate of LUAD is less than 20% (4, 5). Some LUAD with specific mutations can be treated with targeted therapy drugs, but there is a serious problem of drug resistance, and the effects of other treatments such as immunotherapy, radiotherapy, and chemotherapy on LUAD are also very limited (6, 7, 8). Therefore, it is crucial to explore new therapeutic targets for LUAD.

M^6^A is the most abundant mRNA modification in eukaryotes, determines the fate of the modified RNA molecule at the post-transcriptional level, and affects almost all important biological processes (9, 10). The biological function of m^6^A modification largely depends on its "Reader", for example, YTHDC2 (11). YTHDC2 exhibits 3′ → 5′ RNA helicase activity and shares a resemblance in its YTH domain to a recognition pocket for m^6^A. This pocket has been proven to effectively bind m^6^A-modified RNAs in laboratory settings (12). Loss of *Ythdc2* results in male and female infertility in mice (13). Mechanistically, YTHDC2 can affect its bound m^6^A-modified RNA expression by affecting the stability and subsequent translation (11, 14). Our laboratory found that the expression of YTHDC2 is significantly downregulated and correlated with poor prognosis in LUAD. Subsequent experiments confirms that YTHDC2 recognizes m^6^A of solute carrier 7A11 (SLC7A11) and homeobox A13 (HOXA13) mRNA through its YTH domain, resulting in decreases in the expression of SLC7A11, HOXA13, and its downstream SLC3A2 and inhibiting the antioxidant function of LUAD cells (15, 16). Therefore, the decrease of YTHDC2 enhances the antioxidant capacity and malignancy of LUAD cells. However, whether YTHDC2 is related to other cancer-promoting biological phenomena, and more downstream genes and molecular mechanisms of YTHDC2 affects LUAD still need to be explored.

About 0.1 to 0.4% of all adenosines in isolated RNA are m^6^A modified in mammals, accounting for approximately 50% of total methylated ribonucleotides, so m^6^A is a widely occurring modification (17). In the process of tumor formation and development, the regulatory role of m^6^A is also very extensive. m^6^A can regulate various signaling pathways, including PI3K/AKT/mTOR, Hippo/YAP, Wnt, *etc.* (18, 19, 20), and can also regulate various metabolisms including glucose metabolism (21), fatty acid metabolism (22), amino acid metabolism (23), mitochondrial metabolism (24), *etc.* Exosome is a type of small vesicle that is released by cells through endocytosis (25) and plays a crucial role in controlling cell signaling and has the ability to influence tumor microenvironments, as well as the growth and progression of tumors (26). Exosome is important for cell-to-cell communication as it is taken up by target cells and is also secreted by host cells. As a result, exosome serves as essential messengers in facilitating communication between cells (27). In recent years, many studies have involved m^6^A and exosome, but there are still relatively few studies exploring the direct relationship between them. Although the role of m^6^A in regulating tumors is double-sided, there are reports that m^6^A promotes or suppresses tumors (28), but YTHDC2, as an effector molecule after m^6^A, usually plays a role of tumor suppressor (16, 29). In this study, YTHDC2 will be selected as an entry point to explore its impact on exosome. The key downstream target genes of YTHDC2 would be found, and the molecular mechanism would be analyzed.

In this study, we found that the decrease in YTHDC2, a phenomenon that usually occurs in LUAD, led to an increase in exosome content. Through RNA-seq analysis, we identified a series of target genes of YTHDC2, including OASs and IFITs, and analyzed their m^6^A sites and the related mechanism by which their expression was controlled by YTHDC2. We next analyzed YTHDC2, OASs, and IFITs downstream genes that affected exosome content including RAB5A, RAB7, and RAB11A, and the molecular mechanisms were further explained in detail. Because the phenomenon of YTHDC2 decline was more likely to occur in acinar LUAD, the above mechanism might provide a new theoretical basis for the treatment of acinar LUAD.

## Results

### YTHDC2 knockout increases exosome content in LUAD

**Figure 1:**
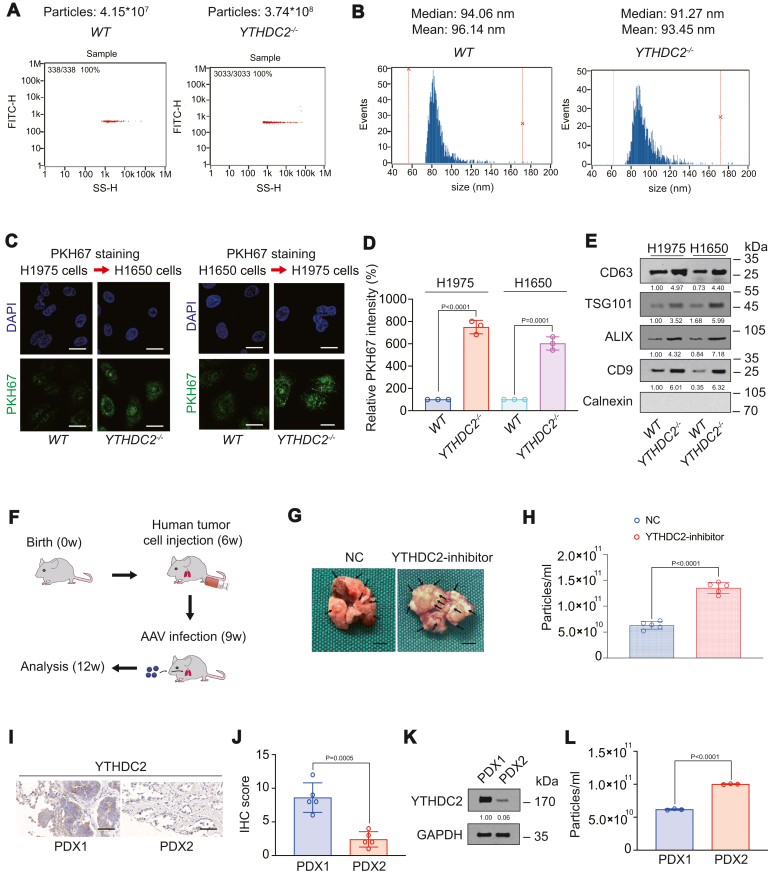
**YTHDC2 knockout increased exosome concentration.***A* and *B*, data from Flow NanoAnalyzer displayed particle concentration (*A*) and size distribution (*B*). *C* and *D*, transfer of exosome between *WT* or *YTHDC2*^*−/−*^ H1975 and H1650 cells. The exosome was marked by PKH67 (*green*) and incubated with H1975 and H1650 cells. Afterward, IF was performed for the detection of exosome. Scale bar represents 20 μm (*C*). The relative PKH67 intensity was measured using ImageJ (*D*). *E*, CD63, TSG101, ALIX, CD9, and Calnexin was measured in exosome extracted from H1975 and H1650 cells. The level of proteins was normalized to that of GAPDH, and the normalized level of proteins in H1975 *WT* cells was arbitrarily set to 1. The number of cells in *WT* and *YTHDC2*^*−/−*^ samples was normalized to the same amount. *F*, flow chart of mouse intrapulmonarily injected H1975 cells and infected with AAV5. *G*, representative images of mouse lungs (*black* arrow indicates tumors). Scale bar represents 2 mm. *H*, particle concentration of plasma in AAV5-infected mouse model analyzed by Flow NanoAnalyzer. *I*–*L*, representative YTHDC2 staining IHC images (*I*) and IHC score (*J*) of PDX#1 and PDX#2 mice (*I*). YTHDC2 expression was also measured by IB. The level of YTHDC2 protein was normalized to that of GAPDH, and the normalized level of YTHDC2 protein in PDX1 was arbitrarily set to 1 (*K*). Particle concentration of mouse plasma was analyzed by Flow NanoAnalyzer (*L*). The mice were euthanized at 36 days after passage. Scale bar represents 100 μm. The data are shown as the mean ± SD from three or five biological replicates. ∗∗*p* < 0.01 indicates statistical significance. Data in *D*, *H*, *J*, and *L* were analyzed by a student’s *t* test.

After knocking out YTHDC2 in H1975 cells, we observed that the number of exosome in the medium increased significantly (Figs. 1*A* and S1, *A*–*D*), while the size of the exosome did not change significantly (Fig. 1*B*). We used the PKH67 staining experiment (30, 31) to confirm that exosome can be delivered between H1650 and H1975 cells (Fig. 1*C*). Moreover, both the intensity of PKH67 and the number of positive cells were significantly higher in *YTHDC2*^*−/−*^ cells than in *WT* cells (Figs. 1, *C* and *D* and S1*E*). In the collected exosome, we could detect markers such as CD63, TSG101, ALIX, and CD9. When the sample was collected at a constant volume, the expression of these markers in *YTHDC2*^*−/−*^ cells increased (Fig. 1*E*). When knocking down YTHDC2 expression using shRNA (Fig. S1, *F* and *G*), we obtained similar results to the CRISPR-based knockout method: inhibition of YTHDC2 expression upregulated exosome content (Fig. S1*H*), the intensity of another exosome dye PKH26 (Fig. S1, *I* and *J*), the number of PKH26 positive cells (Fig. S1*K*), and the expression of CD63, TSG101, ALIX, and CD9 when the exosome sample was collected at a constant volume (Fig. S1*L*). We constructed a model of intrapulmonary injection of H1975 cells and infection with adeno-associated virus 5 (AAV5) to verify the role of reducing YTHDC2 expression. In the week 6, H1975 cells were injected into the lungs of mice, then infected with AAV5-expressing NC or YTHDC2 inhibitor at week 9, and analyzed at week 12 (Fig. 1*F*). We found that YTHDC2 inhibitor increased the number of tumor growth in the lungs of mice and the content of plasma exosome (Figs. 1, *G* and *H* and S1, *M* and *N*). The YTHDC2 inhibitor had similar inhibitory efficiency on YTHDC2 protein in H1975 cells injected into the lungs of nude mice at 3 and 6 weeks after infection (Fig. S1*O*), and its distribution in the lungs was significantly higher than that in other organs (Fig. S1*P*). In addition, higher fold doses of YTHDC2 inhibitor did not affect the survival rate of mice (Fig. S1*Q*). We also constructed a cell-derived xenograft (CDX) model with *WT* or *YTHDC2*^*−/−*^ H1975 cells with or without treatment with GW4869 (exosome biogenesis inhibitor) and found that knockout of YTHDC2 could significantly increase tumor volume and exosome content, and this effect could be reversed by GW4869 (Fig. S1, *E*–*G*). In addition, we constructed two series of patient-derived xenograft (PDX) model with YTHDC2 high expression or low expression and confirmed that YTHDC2 expression was negatively associated with the content of exosome (Fig. 1, *I*–*L*). The above results clarified that YTHDC2 knockout would increase the content of exosome in LUAD.

### Identification of downstream target genes of YTHDC2

**Figure 2:**
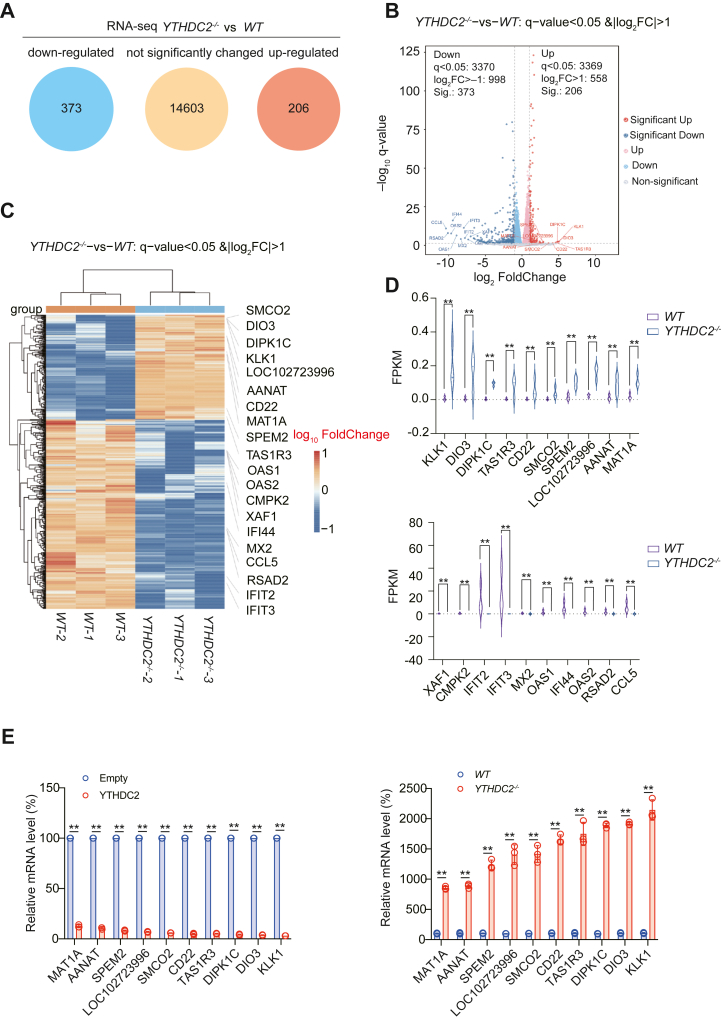
**Identification of YTHDC2 downstream genes by RNA-seq.***A*–*C*, mRNAs affected by YTHDC2 knockout in H1975 cells identified *via* RNA-seq as shown by sector graph (*A*), volcano plot (*B*), and heatmap (*C*). *D*, top 10 upregulated and downregulated mRNAs in RNA-seq as shown by *violin* plot. *E*, top 10 YTHDC2 knockout upregulated mRNAs were validated by qPCR in YTHDC2 overexpressed or knockout H1975 cells. The data are shown as the mean ± SD from three biological replicates. ∗∗*p* < 0.01 indicates statistical significance. Data in *D* and *E* were analyzed by a student’s *t* test.

In order to clarify the molecular mechanism how YTHDC2 knockout to promote exosome secretion, we performed RNA-seq to reveal the downstream genes of YTHDC2. Compared with WT group, a total of 373 significantly decreased genes and 206 significantly increased genes were revealed in *YTHDC2*^*−/−*^ group, while the remaining 14,603 genes were not significantly changed (Fig. 2, *A*–*C*). The present criteria were q-value<0.05 and |log_2_FC|>1. According to the q-value, the top 10 decreased genes were KLK1, DIO3, DIPK1C, TAS1R3, CD22, SMCO2, SPEM2, LOC102723996, AANAT, and MAT1A; and the top 10 increased genes were XAF1, CMPK2, IFIT2, IFIT3, MX2, OAS1, IFI44, OAS2, RSAD2, CCL5 (Fig. 2*D*). The consistency between groups of RNA-seq data is also relatively good, and there is no significant difference in Gene number and total FPKM values between groups (Fig. S2, *A* and *B*). We also validated these YTHDC2 target genes in H1975 cells using qPCR-based method and confirmed that YTHDC2 overexpression promoted, knockout inhibited the expression of CCL5, RSAD2, OAS2, IFI44, OAS1, MX2, IFIT3, IFIT2, CMPK2, IFIT2, and XAF1, while YTHDC2 overexpression inhibited, knockout promoted MAT1A, AANAT, SPEM2, LOC102723996, SMCO2, CD22, TAS1R3, DIPK1C, DIO3, and KLK1 expression (Figs. 2*E* and S2, *C* and *D*).

### YTHDC2 downstream target genes affected exosome content in a YTHDC2-dependent manner

**Figure 3:**
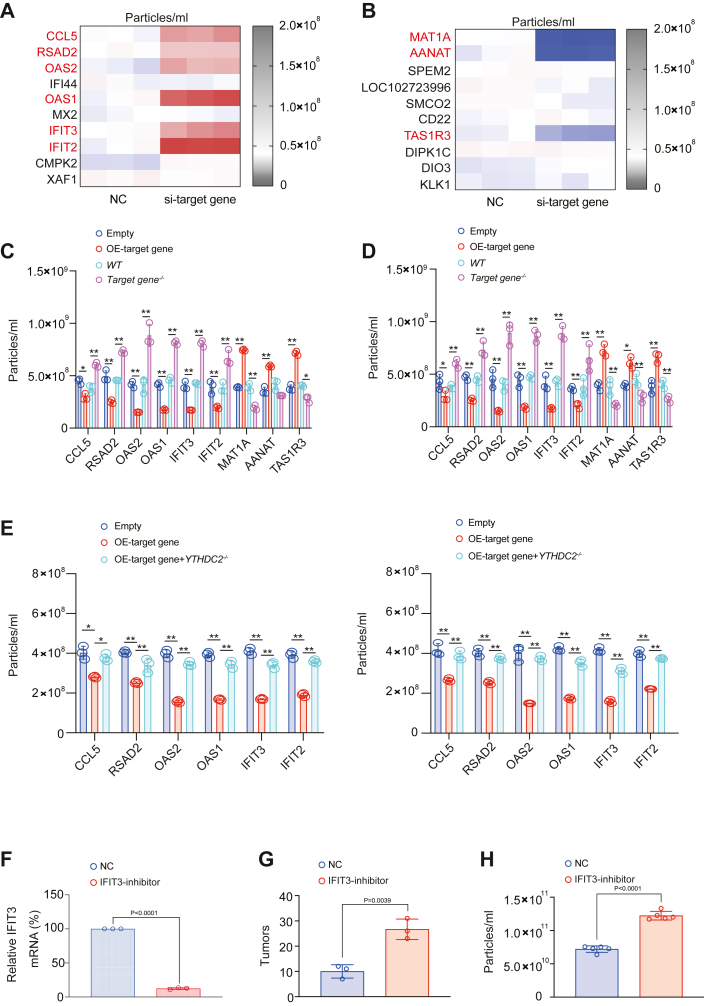
**YTHDC2 downstream target genes could affect exosome concentration.***A*-*B*, the heat map showed which siRNA affected particle concentration in H1975 cells, and the genes that affected particle concentration after being knocked out were marked in *red*. *C* and *D*, particle concentration was measured after indicated target gene overexpression or knockout in H1975 cells. *E*, particle concentration was measured in H1975 and H1650 cells with target gene overexpression with or without YTHDC2 knockout. *F*–*H*, relative IFIT3 mRNA (*F*), tumor number (*G*), and particle concentration (*H*) measured in NC and IFIT3-inhibtor infected mouse model. The cultivation process of the intrapulmonary injection LUAD-bearing and AAV5-infected mouse model refers to Figure 1, *F*–*H*. The data are shown as the mean ± SD from three biological replicates. ∗*p* < 0.05, ∗∗*p* < 0.01 indicates statistical significance. Data in *C*, *D*, *F*, and *H* were analyzed by a student’s *t* test. Data in (*E*) were analyzed by a one-way ANOVA test.

Since we confirmed that YTHDC2 has an effect on the content of exosome in the tumor microenvironment (Figs. 1 and 2), we next explored whether the discovered YTHDC2 target genes have the function for regulating exosome content. First, we designed siRNAs for the top ten upregulated and downregulated genes found by RNA-seq after YTHDC2 knockout (Fig. S3*A*) and confirmed that after knockout of these 20 genes, CCL5, RSAD2, OAS2, OAS1, IFIT3, and IFIT2 could increase the content of exosome, while MAT1A, AANAT, TAS1R3 had the function for decreasing the content of exosome (Figs. 3, *A* and *B* and S3*B*). We further confirmed the function of these genes regulating exosome content through the overexpression and knockout vectors of the above genes (Figs. 3, *C* and *D* and S3*C*) and found that knocking out YTHDC2 reversed the ability of CCL5, RSAD2, OAS2, OAS1, IFIT3, and IFIT2 in reducing the content of exosome, indicating that these genes could reduce the content of exosome through a mechanism related to YTHDC2 (Fig. 3*E*). We also constructed a model of intrapulmonary injection of H1975 cells and infection with AAV5 containing IFIT3 inhibitor and confirmed that knocking down IFIT3 had the function of promoting tumor growth and increasing plasma exosome content (Fig. 3, *F*–*H*). The IFIT3 inhibitor exhibited comparable inhibitory efficacy on the IFIT3 protein in H1975 cells that were injected into the lungs of nude mice at 3 and 6 weeks after infection (Fig. S3*D*), with notably elevated levels of distribution observed in the lungs compared to other organs (Fig. S3*E*). Furthermore, administering higher doses of the IFIT3 inhibitor did not impact the survival rate of the mice (Fig. S3*F*). Altogether, we revealed that some target genes such as OAS1, OAS2, IFIT2, and IFIT3 had the function of regulating exosome content, and this function was closely related to YTHDC2.

### OAS and IFIT mRNAs were m^6^A modified

**Figure 4:**
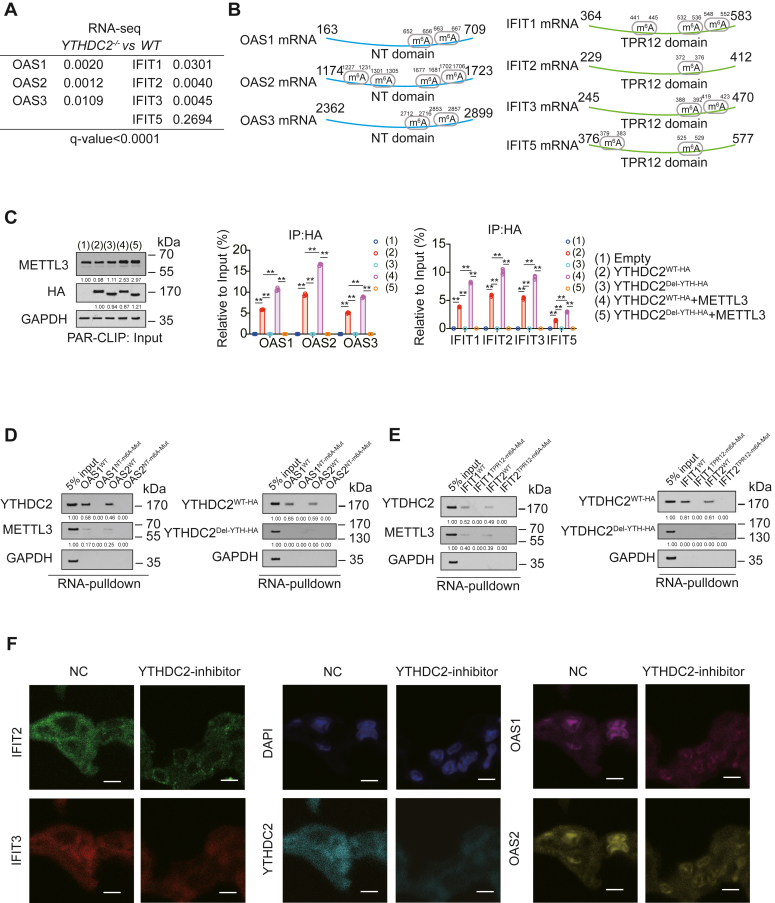
**OAS and IFIT mRNAs were m**^**6**^**A modified.***A*, OAS and IFIT fold changes between groups in RNA-seq. *B*, m^6^A modification sites on OAS NT domain and IFIT TPR12 domain. *C*, PAR-CLIP assay of RNA pulled down by HA-tagged YTHDC2^WT^ or YTHDC2^Del-YTH^ in H1975 cells with or without overexpressing METTL3. The level of proteins was normalized to that of GAPDH. OAS and IFIT mRNA levels in the pulled-down products were also verified by RT-qPCR. *D* and *E*, RNA pull-down experiments in H1975 cells using WT or m^6^A mutant OAS1/2 (*D*) or IFIT1/2 (*E*) probes, as indicated, and the interaction of YTHDC2 and METTL3 was revealed by IB. The level of proteins in 5% input sample was arbitrarily set to 1. *F*, IFIT2, IFIT3, OAS1, and OAS2 expression analyzed by multichannel IF in NC and YTHDC2-inhbitor AAV5-infected mouse with H1975 cell intrapulmonarily injected. Scale bar represents 50 μm. The data are shown as the mean ± SD from three biological replicates. ∗*p* < 0.05, ∗∗*p* < 0.01 indicates statistical significance. Data in (*A*) were analyzed by a student’s *t* test. Data in (*C*) were analyzed by a one-way ANOVA test.

OAS1, OAS2, IFIT2, and IFIT3 were all included in the top ten downregulated genes of *YTHDC2*^*−/−*^ *versus WT*, OAS3, and IFIT5, which were also downregulated after YTHDC2 knockout (Fig. 4*A*). YTHDC2 generally plays a regulatory role after target genes modified with m^6^A. Therefore, we next explored whether m^6^A modification occurred in OASs and IFITs. Through the query on the NCBI website, we found that OASs all have an NT domain, and IFITs all have a TPR12 domain. Through searching for RRACU motif (R for A or G), we found that m^6^A modification sites existed at the NT domain of OAS1-3, the TPR12 domain of IFIT1-3 and IFIT5 (Fig. 4*B*). We also confirmed through photoactivatable ribonucleoside-enhanced crosslinking and immunoprecipitation (PAR-CLIP) experiments that the YTH domain on YTHDC2 was the key domain for binding with m^6^A-modified OAS and IFIT mRNAs, and METTL3 was the key "writer" for this modification (Fig. 4*C*). We constructed reconstituted cells to replace METTL3 with METTL14 and found that METTL14 could not realize the m^6^A modification of OAS1 and IFIT2, indicating that METTL14 was not the key "writer" (Fig. S4, *A* and *B*). Subsequently, we constructed OAS NT domain m^6^A mutation (OAS^NT-m6A-Mut^) and IFIT TPR12 domain m^6^A mutation (IFIT^TPR12-m6A-Mut^) cells according to the m^6^A modification sites marked in Figures 4*B* and S4, *C* and *D*), and it was clear that the m^6^A modification of OASs and IFITs decreased significantly after the m^6^A modification sites were mutated (Fig. S4*E*). We also used RNA pull-down experiments and observed that compared with the WT-probe, the probes of OAS NT domain m^6^A mutation (OAS^NT-m6A-Mut^) and IFIT TPR12 domain m^6^A mutation (IFIT^TPR12-m6A-Mut^) no longer bound to YTHDC2 and METTL3. Moreover, after the YTH domain of YTHDC2 was deleted, it lost the ability to bind to OAS^WT^ and IFIT^WT^ probes (Fig. 4, *D* and *E*). In addition, METTL3 overexpression could affect the total m^6^A modification level, while WT-IFIT, TPR12-m^6^A-mut type, WT-OAS1, and NT-m^6^A-mut type did not significantly affect the total m^6^A modification (Fig. S4*F*). We also confirmed that the expression of IFIT2, IFIT3, OAS1, and OAS2 decreased after YTHDC2 inhibition in AAV5-infected model by multichannel immunofluorescence (IF) (Figs. 4*F* and S4*G*). In conclusion, we identified that OAS and IFIT mRNAs could be m^6^A modified.

### m^6^A modification upregulated OAS and IFIT mRNA stability

**Figure 5:**
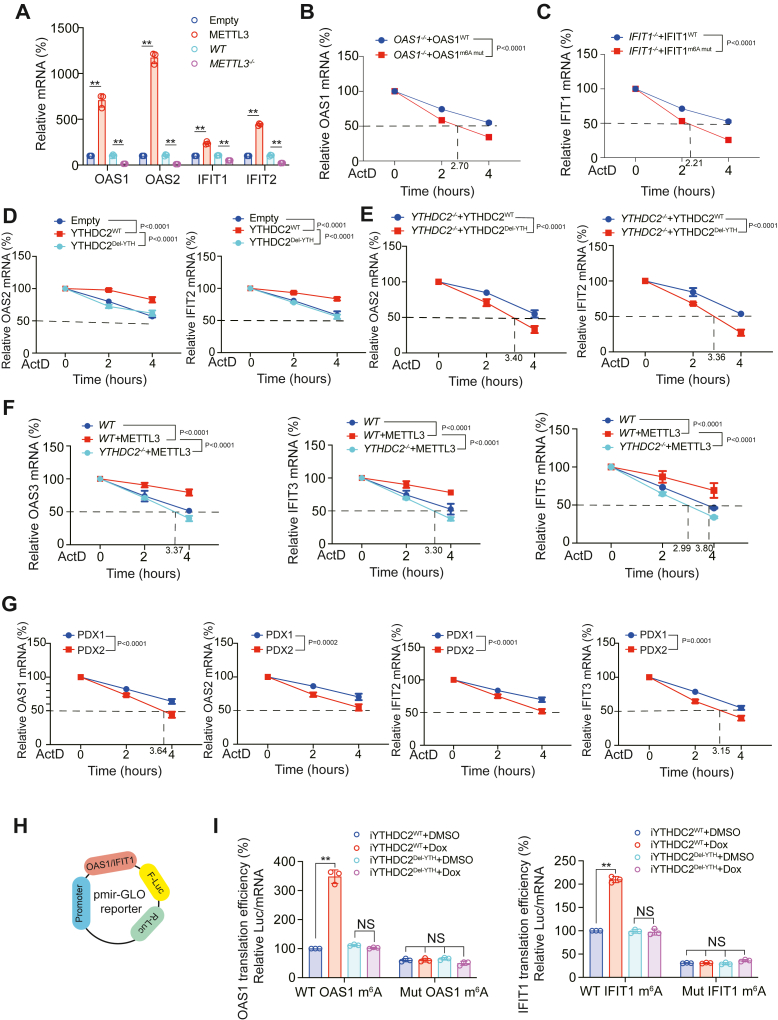
**m**^**6**^**A modification upregulated OAS and IFIT mRNA stability in a YTHDC2-dependent manner.***A*, OAS1, OAS2, IFIT1, and IFIT2 mRNA level analyzed in H1650 cells with METTL3 overexpression or knockout. *B*, OAS1 mRNA stability was analyzed in *OAS1*^*−/−*^ H1650 cells with OAS1 WT or OAS1 m^6^A mut overexpression at indicated time after ActD treatment. *C*, IFIT1 mRNA stability was analyzed in *IFIT1*^*−/−*^ H1650 cells with IFIT1 WT or IFIT1 m^6^A mut overexpression at indicated time after ActD treatment. *D*, OAS2 and IFIT2 mRNA stability was analyzed in H1650 cells with YTHDC2^WT^ or YTHDC2^Del-YTH^ overexpression at indicated time after ActD treatment. *E*, OAS2 and IFIT2 mRNA stability was analyzed in *YTHDC2*^*−/−*^ H1650 cells with YTHDC2^WT^ or YTHDC2^Del-YTH^ overexpression at indicated time after ActD treatment. *F*, OAS3, IFIT3, and IFIT5 mRNA stability was analyzed in H1650 cells with METTL3 overexpression with or without YTHDC2 knockout at indicated time after ActD treatment. *G*, OAS1, OAS2, IFIT2, and IFIT3 mRNA stability was analyzed in primary cells from LUAD tissues constructed for PDX models at indicated time after ActD treatment. *H*, schematic generation strategy for the pmir-Glo luciferase reporters containing OAS1/IFIT1 CDS region. *I*, luciferase activities from the pmir-Glo vector containing WT or m^6^A site mutant OAS1/IFIT1 CDS region were measured in H1650 cells with or without Dox-inducible YTHDC2^WT^ or YTHDC2^Del-YTH^ overexpression with or without Dox treatment. Translation efficiency was calculated as the ratio of luciferase activity to mRNA content. The data are shown as the mean ± SD from three biological replicates. ∗*p* < 0.05, ∗∗*p* < 0.01 indicates statistical significance. Data in (*A*) were analyzed by a student’s *t* test. Data in (*B*–*G*) were analyzed by a two-way ANOVA test. Data in (*I*) were analyzed by a one-way ANOVA test.

M^6^A modifications often influence the stability of target mRNAs (32, 33). We found that METTL3 overexpression upregulated, whereas METTL3 knockdown downregulated the expressions of OAS and IFIT mRNAs (Fig. 5*A*). Compared to OAS1^WT^ and IFIT1^WT^, OAS1^m6A mut^ and IFIT1^m6A mut^ mRNA stability decreased significantly (Fig. 5, *B* and *C*). In addition, YTHDC2 overexpression increased OAS2 and IFIT2 mRNA stability, whereas YTHDC2 YTH domain deletion (YTHDC2^Del-YTH^) did not have this effect (Fig. 5*D*). We also constructed YTHDC2 reconstituted cells, which further confirmed that in YTHDC2^WT^ cells, the mRNA stability of OAS2 and IFIT2 was higher than that of YTHDC2^Del-YTH^ cells (Fig. 5*E*). In addition, we observed that METTL3 upregulated the mRNA stability of OAS3, IFIT3, and IFIT5, and this effect was reversed by knockout of YTHDC2 (Fig. 5*F*). In the primary cells from LUAD tissues constructed for PDX model (previously described in Fig. 1, *I*–*L*), we also observed that stability of OAS1, OAS2, IFIT2, and IFIT3 mRNA was high in PDX1 (high YTHDC2 level) compared to PDX2 (low YTHDC2 level) (Fig. 5*G*). Altogether, these results indicated that METTL3-mediated m^6^A modification upregulated the stabilities of OAS and IFIT mRNAs in a YTHDC2-dependent manner.

### m^6^A modification stimulated translation of OASs and IFITs

To investigate whether YTHDC2-mediated m^6^A modification affects translation of OASs and IFITs, the CDS region of OAS1 and IFIT1 mRNA with or without m^6^A site mutation was cloned into the pmir-GLO plasmid (Fig. 5*H*). Using a Dox-inducible YTHDC2 (iYTHDC2) plasmid, we observed that YTHDC2^WT^ could upregulate the translational efficiencies of OAS1 and IFIT1 in the presence of m^6^A sites, but YTHDC2^Del-YTH^ had no such effect. When the m^6^A site was mutated, YTHDC2 no longer regulated the translational efficiencies of OAS1 and IFIT1 (Fig. 5*I*). XRN2 and EXOSC10 are two major exonucleases responsible for 5′ -3′ and 3′ -5′ exonucleolytic activity, respectively (16, 34, 35). Deletion of XRN2, but not EXOSC10, reversed the effect of knocking out METTL3 on inhibiting the level of OAS and IFIT mRNAs (Fig. S5*A*). In addition, deletion of XRN2 could reverse the decrease in mRNA stability caused by m^6^A site mutations in OAS2 and IFIT2 (Fig. S5, *B* and *C*). Therefore, we believed that m^6^A modification promoted the translation of OAS and IFIT mRNAs in a YTHDC2-dependent manner.

### OAS and IFIT promoted malignant phenotype in LUAD cells

Next, we explored the effects of OASs and IFITs on the malignant phenotype of LUAD cells and whether they were related to YTHDC2. We found that the decrease in cell viability (Fig. S6*A*), colony formation (Fig. S6, *C* and *D*), exosome content (Fig. S6*L*), as well as the increase in autophagy level (Fig. S6*I*) and 4-HNE level (Fig. S6*J*) caused by overexpression of OAS1, IFIT1, OAS3, and IFIT3 was reversed by the knockout of YTHDC2; but caspase 3/7 activity was not affected by the above treatments (Fig. S6*G*). The increase in cell viability (Fig. S6*B*), colony formation (Fig. S6, *E* and *F*), exosome content (Fig. S6*M*), as well as the decrease in autophagy level (Fig. S6*I*) and 4-HNE level (Fig. S6*K*) caused by the knockout of OAS2, IFIT2, OAS1, and IFIT5 could be reversed by the addition of the exosome inhibitor GW4869; similarly, caspase 3/7 activity was not affected by the above treatments (Fig. S6*H*). The above results indicate that OASs and IFITs may induce oxidative stress-related death caused by peroxides such as 4-HNE but do not induce caspase-related apoptosis, and this ability was dependent on their upstream YTHDC2 and might be accomplished by downregulating the content of exosome.

### Identification of OAS and IFIT target genes for promoting exosome

**Figure 6:**
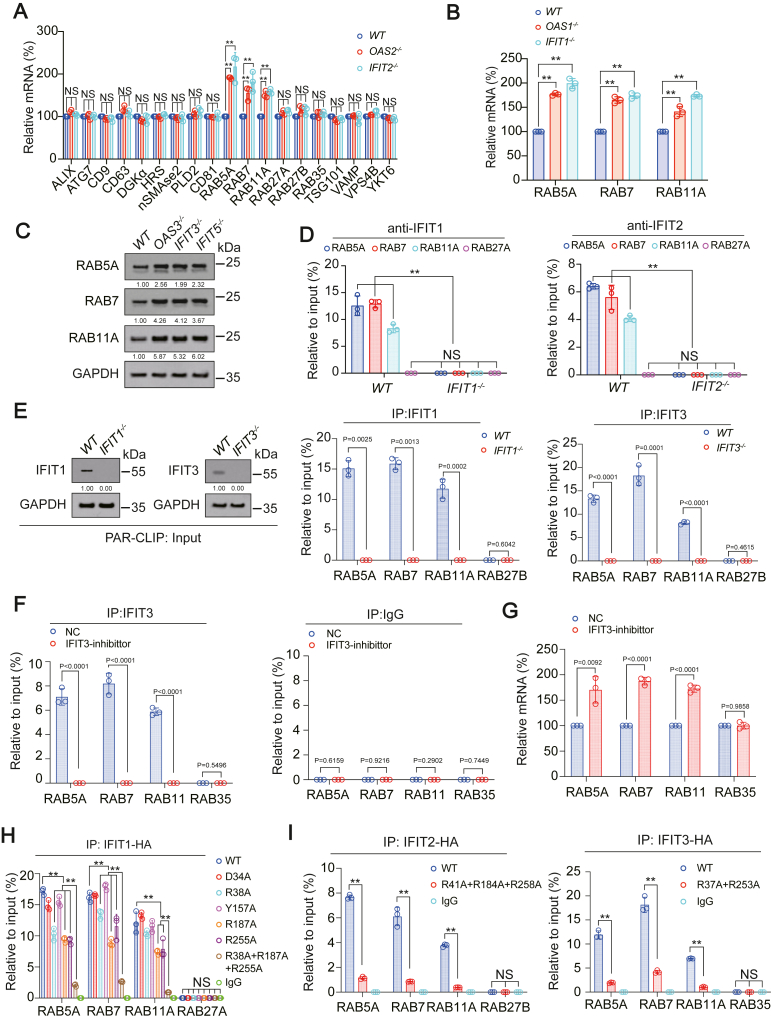
**IFIT reduced the expression of exosome-promoting genes RAB5A, RAB7, and RAB11A.***A*, indicated mRNA was measured in H1975 cells with OAS2 or IFIT2 knockout. *B*, RAB5A, RAB7, and RAB11A mRNA was measured in H1975 cells with or without OAS1 or IFIT1 knockout. *C*, RAB5A, RAB7, and RAB11A protein levels were analyzed by IB in H1975 cells with or without OAS3, IFIT3, or IFIT5 knockout. The level of proteins was normalized to that of GAPDH, and the normalized level of proteins in *WT* cells was arbitrarily set to 1. *D*, RNA-IP assay of RNA pulled down by anti-IFIT1 or anti-IFIT2 in H1975 cells with or without IFIT1 or IFIT2 knockout. RAB5A, RAB7, RAB11A, and RAB27A mRNA levels in the pulled down products were also verified by RT-qPCR. *E*, PAR-CLIP assay of RNA pulled down by anti-IFIT1 or anti-IFIT3 in H1975 cells with or without IFIT1 or IFIT3 knockout. The level of proteins was normalized to that of GAPDH, and the normalized level of proteins in *WT* cells was arbitrarily set to 1. RAB5A, RAB7, RAB11A, and RAB27B mRNA levels in the pulled-down products were also verified by RT-qPCR. *F*, RNA-IP assay of RNA pulled down by anti-IFIT3 or IgG in intrapulmonary injection LUAD-bearing and NC or IFIT3-inhibitor AAV5 infected mouse model. RAB5A, RAB7, RAB11A, and RAB35 mRNA levels in the pulled down products were also verified by RT-qPCR. *G*, RAB5A, RAB7, RAB11A, and RAB35 mRNA level in intrapulmonary injection LUAD-bearing and NC or IFIT3-inhibitor AAV5 infected mouse model. *H*–*J*, RNA-IP assay of RNA pulled down by HA-tagged IFIT1 (*H*), IFIT2 (*I*), or IFIT3 (*J*) with indicated site mutated in H1975 cells. RAB5A, RAB7, RAB11A, RAB27A, RAB27B, or RAB35 mRNA levels in the pulled down products were also verified by RT-qPCR. The data are shown as the mean ± SD from three biological replicates. ∗∗*p* < 0.01 indicates statistical significance. NS, non-significance. Data in *A*, *B*, *D*, *H*, and *I* were analyzed by a one-way ANOVA test. Data in E-G were analyzed by a student’s *t* test.

In order to find the target genes of OASs and IFITs that promoted exosome, we knocked out OAS2 and IFIT2 in H1975 cells respectively, screened a series of mRNAs related to exosome biogenesis, transport and release by qPCR, and observed whether their expression was affected by OAS2 and IFIT2. We found that the expressions of RAB5A, RAB7, and RAB11A were significantly increased after OAS2 and IFIT2 knockout (Fig. 6*A*). In addition, we also verified that knocking out OAS1 or IFIT1 significantly increased the mRNA level of RAB5A, RAB7, and RAB11A (Fig. 6*B*), while knocking out OAS3, IFIT3, and IFIT5 significantly increased the protein level of RAB5A, RAB7, and RAB11A (Fig. 6*C*). The above results confirmed that RAB5A, RAB7, and RAB11A were the target genes of OASs and IFITs in promoting exosome.

### Mechanism onhow IFITs triggered the degradation of target genes

IFITs can bind to other mRNAs and inhibit their translation (36, 37). Therefore, we speculated that IFITs bound to RAB5A, RAB7, and RAB11A mRNAs and then suppressed their translation. Through RNA-immunoprecipitation (RIP) experiments, we found that both IFIT1 and IFIT2 proteins bound to the mRNA of RAB5A, RAB7, and RAB11A but not to RAB27A (Figs. 6*D* and S7*A*). In addition, PAR-CLIP experiments also confirmed that IFIT1 and IFIT3 proteins bound to the mRNA of RAB5A, RAB7, and RAB11A but not to RAB27B (Fig. 6*E*). In the established AAV5-infected model (previously described in Fig. 3, *F*–*H*), we also observed that compared to the NC group, IFIT3 inhibitor inhibited IFIT3 interaction with RAB5A, RAB7, and RAB11A mRNA and upregulated RAB5A, RAB7, and RAB11A mRNA level; there was no binding between IFIT3 and RAB35, and IFIT3 inhibitor did not affect the mRNA level of RAB35 (Fig. 6, *F* and *G*). Next, we constructed pmir-GLO reporters containing RAB5A, RAB7, RAB11A, or RAB27B ORFs to explore the mechanism how IFITs affected the translation of RAB5A, RAB7, RAB11A, and RAB27B (Fig. S7*B*). We found that overexpression of IFIT1 significantly inhibited, whereas knockdown of IFIT2 and IFIT3 significantly promoted the translational activities of RAB5A, RAB7, and RAB11A; but their overexpression or knockout did not affect the translational activities of RAB27B (Fig. S7*C*). Existing studies have shown that IFIT3 can bind and stabilize IFIT1 and promote the combination of IFIT1 and mRNA. Moreover, IFIT2 and IFIT3 can form a more stable dimer with a stronger function of promoting IFIT1 (36). Indeed, we could find that on the basis of IFIT1 overexpression, IFIT2 or IFIT3 further reduced the translation activity of RAB5A, RAB7, and RAB11A, and the combined overexpression of IFIT1, IFIT2, and IFIT3 inhibited the translation activity of RAB5A, RAB7, and RAB11A to the greatest extent; but RAB27B was not affected by the overexpression of IFIT1/2/3 (Fig. S7*D*). According to previous literature reports, D34, R38, Y157, R187, R255 sites of IFIT1 are related to its binding to mRNA (37). Therefore, we constructed the corresponding mutants of IFIT1, and we found that R38A, R187A, and R255A could indeed inhibit the binding of IFIT1 to RAB5A, RAB7, and RAB11A mRNA, while D34A and Y157A had no such effect, and the R38A+R187A+R255A combined mutation had the most obvious effect on inhibiting the binding to RAB5A, RAB7, and RAB11A mRNA (Fig. 6*H*). We also mutated R sites at similar positions on IFIT2 and IFIT3 and found that the combined mutation of IFIT2 R41A+R184A+R258A and the combined mutation of IFIT3 R37A+R253A could inhibit their binding to RAB5A, RAB7, and RAB11A mRNA (Fig. 6*I*). We also found that the combined mutation of R38A+R187A+R255A of IFIT1 could reverse the decreased protein expression of RAB5A, RAB7, and RAB11A induced by WT-IFIT1, while the combined mutation of R41A+R184A+R258A could reverse the decreased protein expression of RAB5A, RAB7, and RAB11A induced by WT-IFIT2 (Fig. S7*E*). Collectively, we summarized the molecular mechanism of IFITs inhibiting the translation of RAB5A, RAB7, and RAB11A by binding to their mRNAs and analyzed the key sites on IFITs associated with these bindings.

### Mechanism of OASs leading to target gene degradation

**Figure 7:**
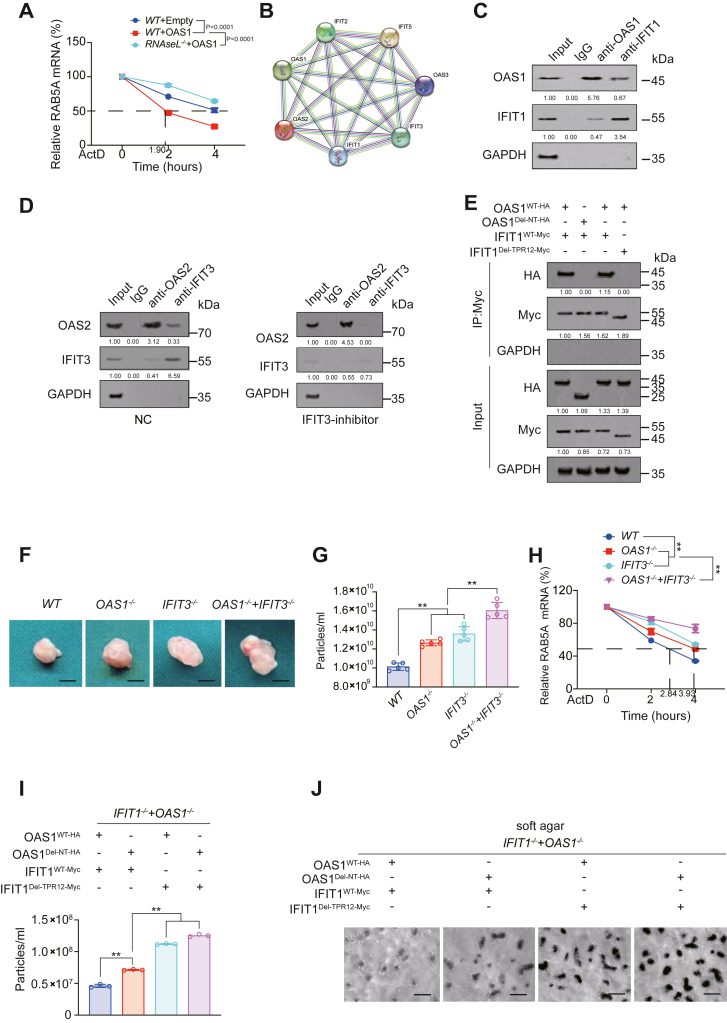
**IFIT and OAS synergistically inhibited RAB5A, RAB7, and RAB11A.***A*, RAB5A mRNA stability was analyzed in H1975 cells with OAS1 overexpression with or without RNAseL knockout at indicated time after ActD treatment. *B*, interaction among IFIT and OAS proteins predicted by String. *C*, co-IP experiments were performed using anti-OAS1 or anti-IFIT1 antibodies in H1975 cells. IgG-immunoprecipitated used samples were analyzed in parallel. Co-immunoprecipitated OAS1 and IFIT1 expression was measured. The level of proteins in Input sample was arbitrarily set to 1. *D*, co-IP experiments were performed using anti-OAS2 or anti-IFIT3 antibodies in intrapulmonary injection LUAD-bearing and NC or IFIT3-inhibitor AAV-infected mouse model. IgG-immunoprecipitated used samples were analyzed in parallel. Co-immunoprecipitated OAS2 and IFIT3 expression was measured. The level of proteins in input sample was arbitrarily set to 1. *E*, co-IP experiments were performed using anti-Myc antibodies in H1975 cells with or without OAS1^WT-HA^, OAS1^Del-NT-HA^, IFIT1 ^WT-Myc^, or IFIT1^Del-TPR12-Myc^ overexpression. The level of proteins in OAS1^WT-HA^ and IFIT1 ^WT-Myc^ overexpression sample was arbitrarily set to 1. *F*–*H*, represented images (*F*), particle concentration (*G*), and RAB5A stability (*H*) for xenograft tumor formed by *WT*, *OAS1*^*−/−*^, *IFIT3*^*−/−*^, *OAS1*^*−/−*^ + *IFIT3*^*−/−*^ H1975 cells. Scale bar represents 5 mm. *I*, particle concentration was analyzed in IFIT1- and OAS1-reconstituted H1975 cells with OAS1^WT-HA^, OAS1^Del-NT-HA^, IFIT1 ^WT-Myc^, or IFIT1^Del-TPR12-Myc^ overexpression. *J*, representative images for soft agar colony formation assay in IFIT1- and OAS1-reconstituted H1975 cells with OAS1^WT-HA^, OAS1^Del-NT-HA^, IFIT1 ^WT-Myc^, or IFIT1^Del-TPR12-Myc^ overexpression. Scale bar represents 100 μm. The data are shown as the mean ± SD from three biological replicates. ∗*p* < 0.05, ∗∗*p* < 0.01 indicates statistical significance. Data in A, H were analyzed by a two-way ANOVA test. Data in *G* and *I* were analyzed by a one-way ANOVA test.

OASs can cause RNA degradation through RNAseL (38). We also found that OAS1-induced decreased mRNA stability of RAB5A, RAB7, and RAB11A could be reversed by knockout of RNAseL (Figs. 7*A* and S8*A*). It was confirmed that RNAseL could be the key factor for OASs to promote the degradation of RAB5A, RAB7, and RAB11A mRNA.

### Mechanism of exosome inhibition by the combination of IFITs and OASs

In order to explore whether IFITs and OASs had a joint regulation mechanism for exosome, we first predicted the mutual binding between IFITs and OASs using the STRING website (https://string-db.org) and found that there was indeed a close mutual binding between OASs and IFITs (Fig. 7*B*). Co-immunoprecipitation (Co-IP) experiments proved the binding between OAS1 and IFIT1, between OAS2 and IFIT2, and between OAS3 and IFIT3 (Figs. 7*C* and S8*B*). The interaction between OAS2 and IFIT3 was also observed in the established AAV5-infected model (previously described in Figs. 3, *F*–*H* and 7*D*). Because the NT domain and TPR12 domain were the domains we found to be important for the functions of OASs and IFITs, respectively, we further explored whether these two domains were crucial for the combination between OASs and IFITs. We found that the interaction between OAS1 and IFIT1, OAS3 and IFIT3, and OAS2 and IFIT5 were dependent on the NT domain of OAS and the TPR12 domain of IFIT (Figs. 7*E* and S8*C*). OAS1 and OAS2 further enhanced the ability of IFIT1 and IFIT2 to promote the degradation of RAB5A and RAB7, respectively (Fig. S8, *D* and *E*). In addition, IFIT1 knockout and IFIT3 knockout could reverse the ability of OAS1 and OAS3 to degrade RAB5A and RAB11A mRNA, respectively; whereas OAS1 knockout and OAS3 knockout could not significantly reverse the ability of IFIT1 and IFIT3 to degrade RAB7 and RAB11 A mRNA, respectively (Fig. S8, *F*–*I*). The above results indicated that IFITs acted as the upstream of OASs to promote degradation. We also constructed a new series CDX model including *WT*, *OAS1*^*−/−*^, *IFIT3*^*−/−*^, *OAS1*^*−/−*^ *+ IFIT3*^*−/−*^ group (Fig. 7*F*). We observed that knockout of OAS1 or IFIT3 could increase tumor volume (Fig. S8*J*), exosome content (Fig. 7*G*), and mRNA stability of RAB5A, RAB7, and RAB11A (Figs. 7*H* and S8, *K* and *L*), and combined knockout of OAS1 and IFIT3 further enhanced the above effects (Figs. 7, *F*–*H* and S8, *J*–*L*). These results in CDX model further demonstrated the synergistic effect of OASs and IFITs on the regulation of target genes such as RAB5A, RAB7, and RAB11A. Additionally, it was verified by mutant experiments that both the NT domain of OAS and the TPR12 domain of IFIT were crucial for OASs and IFITs to inhibit exosome content (Fig. 7*I*) and colony formation in an exosome-dependent manner (Figs. 7*J* and S8, *M* and *N*), which suggested that synergistic effect of OASs and IFITs might inhibit tumor growth.

### The mechanism verified A549 and H1299 cell lines

In KRAS mutant cell lines A549 and non KRAS, EGFR mutant cell lines H1299 cells, we verified that YTHDC2 knockout increased the exosome content (Fig. S9, *A* and *B*); target genes revealed by RNA-seq including MAT1A, AANAT, SPEM2, LOC102723996, SMCO2, CD22, TAS1R3, CCL5, RSAD2, OAS2, IFI44, OAS1, MX2, IFIT3 were affected by the change of YTHDC2 expression (Fig. S9, *C* and *D*); OAS1, OAS2, IFIT2, and IFIT3 inhibited exosome content and these effects could be reversed by YTHDC2 knockout (Fig. S9, *E* and *F*); METTL3 knockout could significantly inhibit m^6^A modification of OAS2, IFIT2, OAS3, and IFIT3 (Fig. S9, *G* and *H*); compared with the WT-probe, the probes of OAS^NT-m6A-Mut^ an IFIT^TPR12-m6A-Mut^ no longer bound to YTHDC2 and METTL3 (Fig. S9*I*); after the YTH domain of YTHDC2 was deleted, it lost the ability to bind to IFIT^WT^ probes (Fig. S9*J*); compared to OAS1^WT^, OAS1^m6A mut^ mRNA stability decreased significantly (Fig. S9*K*); in YTHDC2^WT^ cells, the mRNA stability IFIT3 was higher than that of YTHDC2^Del-YTH^ cells (Fig. S9*L*); YTHDC2^WT^ could upregulate the translational efficiencies of OAS1 and IFIT1 in the presence of m^6^A sites, but YTHDC2^Del-YTH^ had no such effect; when the m^6^A site was mutated, YTHDC2 no longer regulated the translational efficiencies of OAS1 and IFIT1 (Fig. S9*M*); the decrease in cell viability and colony formation, as well as the increase in 4-HNE level caused by the overexpression of OAS2 was reversed by knockout of YTHDC2, and the increase in cell viability and colony formation, as well as the decrease in 4-HNE level caused by the knockout of IFIT3 could be reversed by the addition of the exosome inhibitor GW4869, but caspase 3/7 activity was not influenced by the above treatment (Fig. S9, *N*–*V*); knockout of OAS1, IFIT1, OAS2, and IFIT2 could significantly induce the mRNA level of RAB5A, RAB7, and RAB11A, but not influence the level of RAB27B (Fig. S9*W*); both IFIT1 and IFIT2 proteins bound to the mRNA of RAB5A, RAB7, and RAB11A but not to the mRNA of RAB27A (Fig. S9, *X* and *Y*); overexpression of IFIT1 significantly inhibited, whereas knockdown of IFIT2 and IFIT3 significantly promoted the translational activities of RAB5A, RAB7, and RAB11A (Fig. S9*Z*); on the basis of IFIT1 overexpression, IFIT2 or IFIT3 further reduced the translation activity of RAB5A, RAB7, and RAB11A, and the combined overexpression of IFIT1, IFIT2, and IFIT3 inhibited the translation activity of RAB5A, RAB7, and RAB11A to the greatest extent (Fig. S9*A*); combined mutation of IFIT1 R38A+R187A+R255A and IFIT2 R41A+R184A+R258A could inhibit their binding to RAB5A, RAB7, and RAB11A mRNA (Fig. S9*B*); OAS1-induced decreased mRNA stability of RAB5A and RAB7 could be reversed by knockout of RNAseL (Fig. S9, *C* and *D*); the interaction between OAS1 and IFIT1 was dependent on the NT domain of OAS and the TPR12 domain of IFIT (Fig. S9, *E* and *F*); OAS1 and OAS2 further enhanced the ability of IFIT1 and IFIT2 to promote the degradation of RAB5A and RAB7, respectively (Fig. S9, *G* and *H*); both the NT domain of OAS and the TPR12 domain of IFIT were crucial for OASs and IFITs to inhibit exosome-dependent colony formation (Fig. S9*I*). These data suggested that the m^6^A reader YTHDC2 upregulates exosome content *via* inhibiting IFIT and OAS family members found in H1975 and H1650 cell lines, which also exists in KRAS mutant cell lines A549 and non KRAS, EGFR mutant cell lines H1299.

### Clinical significance of YTHDC2 and its downstream factors

**Figure 8:**
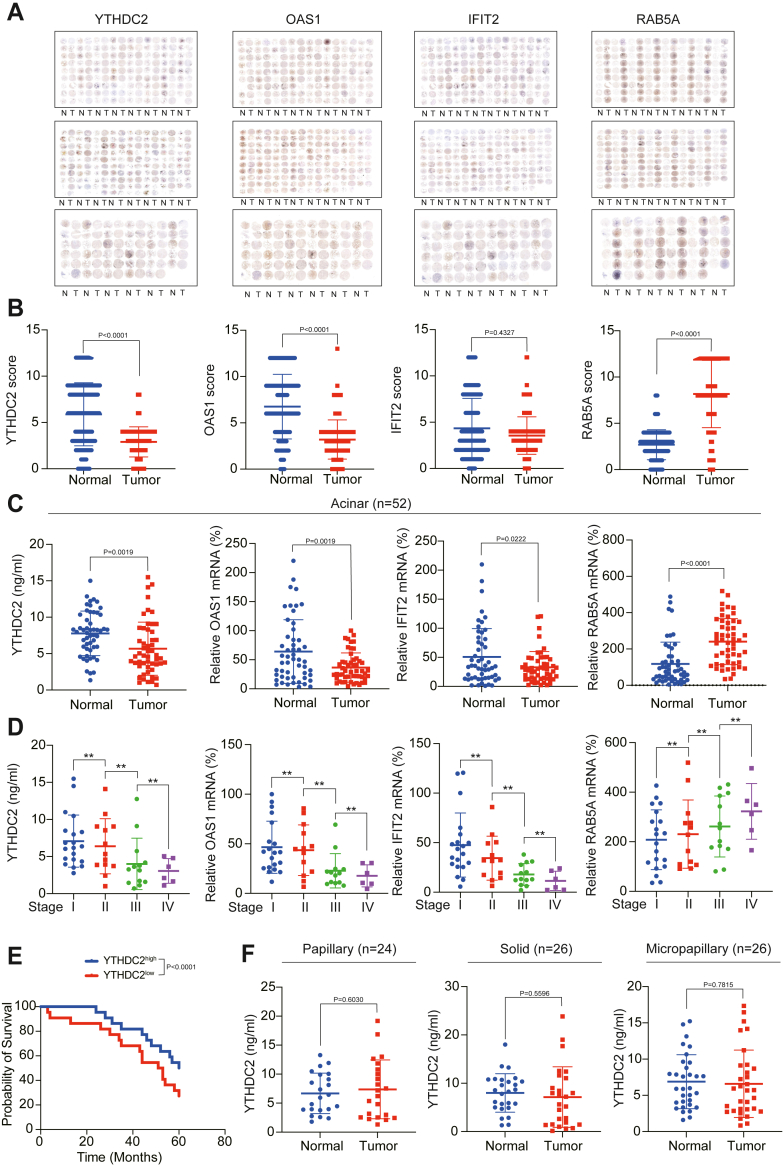
**Clinical significance of YTHDC2-related exosome-stimulating system in LUAD and its acinar subtype.***A* and *B*, immunochemistry stain for LUAD tissue microarray slides using antibodies against YTHDC2, OAS1, IFIT2, and RAB5A. IHC score was displayed in (*B*). *C* and *D*, YTHDC2 protein level and OAS1, IFIT2, and RAB5A mRNA levels were measured in acinar subtype LUAD (*C*). The results were further presented according to the tumor stage (*D*). *E*, Survival analysis in acinar subtype LUAD. Patients were divided into two groups according to the level of YTHDC2 expression. *F*, YTHDC2 protein level were measured in papillary, solid, and micropapillary subtype LUAD. ∗∗*p* < 0.01 indicates statistical significance. NS, non-significance. Data in *B*, *C*, and *F* were analyzed by a student’s *t* test. Data in *D* were analyzed by a one-way ANOVA test. Data in E were analyzed by a log-rank test.

In order to further clarify the clinical significance of the axis in which YTHDC2 regulates exosome content revealed in this study, we used four antibodies against YTHDC2, OAS1, IFIT2, and RAB5A to stain tissue microarrays containing 181 cases of LUAD and adjacent normal samples and confirmed that the corresponding bands could be detected in WT cell lines, but not in knockout cell lines (Fig. S10*A*). The results showed that the expressions of YTHDC2, OAS1, and IFIT2 were all reduced in tumor tissues, whereas the expression of RAB5A was increased in tumor tissues (Fig. 8, *A* and *B*). However, after further analysis, we found that the expression of YTHDC2, OAS1, IFIT2, and RAB5A had no significant relationship with the stage of tumor tissue (Fig. S10*B*), the patient's smoking habits (Fig. S10*C*), the presence of EGFR mutations (Fig. S10*D*), and whether the patient received preoperative treatment (including targeted therapy, immunotherapy, chemoradiotherapy, and so on) (Fig. S10*E*). According to the previous studies, acinar LUAD are more likely to exhibit YTHDC2 downregulation (16). Therefore, we further analyzed the clinical significance of YTHDC2 and its downstream factors based on pathological classification. We found that in acinar LUAD, YTHDC2, OAS1, and IFIT2 were also reduced and RAB5A was increased (Fig. 8*C*), and there was a significant correlation between their expressions (Fig. S10*F*). In addition, their changing trends were related to tumor stage (Fig. 8*D*). Moreover, in acinar LUAD, low YTHDC2 expression indicated poor patient survival rate (Fig. 8*E*). However, even in acinar LUAD, the expression of YTHDC2 was not associated with smoking habits, EGFR mutation status, or whether the patient received preoperative treatment (Fig. S10*G*). Moreover, in other types of LUAD (including papillary, solid, and micropapillary), YTHDC2 expression did not differ between tumor and adjacent normal tissues (Fig. 8*F*), and the expression of YTHDC2 was not associated with EGFR mutation status, smoking habits, tumor stage, or whether the patient received preoperative treatment (Fig. S10, *H*–*J*). Based on the above results, we believed that the activation of the low YTHDC2-related exosome content-increasing system in LUAD was acinar-type–specific.

## Discussion

M^6^A and exosome are two fields that have been researched very hot recently. Researches on the correlation between these two fields have been reported before, which are mainly divided into the following two types: (1) substances in exosome affect the overall m^6^A modification, such as noncoding RNA in exosome exercising the function of ceRNA, promoting the expression of m^6^A-related molecules, or microRNA in exosome directly inhibiting the expression of m^6^A-related molecules (2, 39, 40). m^6^A modification occurs and directly affects the function of exosome-containing noncoding RNA (41, 42). However, the effect of m^6^A on exosome has not been reported before. This study has chosen a novel perspective: firstly, it was confirmed that the exosome content was increased after YTHDC2 knockout, and then a series of target genes of YTHDC2 were screened by RNA-seq. Among them, the OAS family and IFIT family were then focused, and it was clear that their degradation effect was enhanced after m^6^A modification and instead promoted the expression of exosome transport and release genes including RAB5A, RAB7, and RAB11A that were originally inhibited by them. Of course, the increase of exosome content caused by YTHDC2 knockdown promoted the malignant phenotype of LUAD cells, which was consistent with our previous finding that the decrease of YTHDC2 promotes the antioxidant capacity of LUAD cells and promotes tumor progression (15, 16). However, the m^6^A reader is not limited to YTHDC2. After m^6^A modification, other readers might exhibit different functions from YTHDC2, such as inhibiting exosome. For example, YTHDF1 often exhibits cancer-promoting effects in tumors (43, 44). Therefore, the effects of other types of readers on exosome secretion after m^6^A modification and the malignant phenotype of LUAD still need to be further explored.

We found through RNA-seq that after YTHDC2 was knocked out, the expressions of IFITs and OASs were significantly decreased, which indicated that IFITs and OASs were important downstream genes of YTHDC2, and we verified that IFITs and OASs could decline the expression of RAB5A, RAB7, and RAB11A. Both IFITs and OASs have been shown to play a role in viral infection in previous studies (37, 45). IFITs contain five paralogs including IFIT1, IFIT1B, IFIT2, IFIT3, and IFIT5. They take on crucial roles in obstructing viral replication by directly binding the 5′end of viral RNA (46). For example, IFIT1 can compete with eIF4F to selectively bind and sequester vial Cap0-mRNA, resulting in its translational inhibition (36). IFIT1 and IFIT5 possess the capability to detect uncapped viral triphosphate-RNA, which is an additional indicator of infection (47). This recognition ability allows them to potentially impede the replication of certain negative-sense ss RNA viruses (47). In this study, we found that IFITs could recognize mRNAs of RAB5A, RAB7, and RAB11A and inhibit their stability and subsequent translation. Similar to the previous results, our findings further confirmed that IFITs had RNA binding ability and inhibited the translation of target RNA, but it also further expanded the findings that IFITs bound to not only viral RNA but also normal human RNA. The OASs can recognize RNA produced during viral infection and then promote dimerization and activation of latent endoribonuclease RNase L. Activated RNase L can further degrade viral and cellular RNA, including ribosomal RNA, transfer RNA, and specific messenger RNA transcripts (45). In this study, we also found that OAS led to the degradation of downstream RAB5A, RAB7, and RAB11A mRNAs through RNAseL. In addition, we innovatively found that there is an interaction between IFITs and OASs, and IFITs acts as the upstream of OASs to reduce the expression of RAB5A, RAB7, and RAB11A.

In addition to the negative correlation between YTHDC2 and antioxidant capacity of LUAD cells reported by our Lab (15, 16), some other studies have shown that YTHDC2 can affect the malignant phenotype of LUAD cells. For example, overexpression of YTHDC2 inhibits proliferation, migration, and angiogenesis of LUAD cells, as well as growth of LUAD cells in nude mice through long noncoding ZNRD1-AS1 and CYLD/NF-κB signaling pathways; and high expression of YTHDC2 has a better prognosis in LUAD patients (29, 48). The m^6^A demethylase ALKBH5 regulates macrophage M2 polarization through CDCA4 to promote the development of LUAD; and this effect can be reversed by the overexpression of YTHDC2 (49). The above results indicate that overexpression of YTHDC2 may be a means of treating LUAD. If there is a drug that can directly or indirectly upregulate the expression of YTHDC2 or if exogenous YTHDC2 is introduced through gene therapy, it may have a therapeutic effect on LUAD.

Acinar subtype is the most common pathological subtype of LUAD, and previous studies have shown that acinar LUAD subtype cases exhibited significant YTHDC2 downregulation (15, 16). In this study, we excluded the obvious decrease in YTHDC2 in several other types of LUAD, such as papillary, solid, and micropapillary, confirming that the decrease in YTHDC2 is a unique phenomenon of acinar LUAD subtype. In previous studies, since ferroptosis-stimulating targets such as SLC7A11 was highly expressed, pro-ferroptosis factors can induce significant ferroptosis for acinar LUAD subtype (15, 16). Coupled with the axis that promotes exosome expression discovered in this study, it provides two strategies for treating acinar LUAD subtype with low YTHDC2 expression. Perhaps these two strategies can be combined into one to facilitate the precise treatment of lung cancer.

In conclusion, we discovered a mechanism by which low YTHDC2 expression in LUAD promoted exosome by downregulating OASs and IFITs. This mechanism might provide an important theoretical basis for the treatment of acinar LUAD subtype with low YTHDC2 expression.

## Experimental procedures

### Cell culture

Established human LUAD A549, H1299, H1650, and H1975 cell lines were purchased from Fuheng Biotechnology and validated by short tandem repeat analysis. Cells were cultured in Dulbecco’s modified eagle medium (Hyclone) supplemented with 10% fetal bovine serum (Sage Creation Science. Co. Ltd) and 1% penicillin/streptomycin (Hyclone). GW4869 (Sigma) was used to treat cells.

### Animals and tissue samples

All athymic nude mice (6-week-old) were purchased from Jiesijie and bred in specific pathogen-free animal facilities. All animal experiments were approved by the Animal Care Committee of Shanghai Chest Hospital.

To generate routine CDX mouse models, established H1975 cells (initial 5 × 10^6^) were subcutaneously injected into the bilateral dorsal flank of athymic nude mice. The mice were euthanized by cervical dislocation at 36 days after injection.

To generate H1975 cell-implanted intrapulmonary LUAD mice, 6-weeks athymic nude mice were intrapulmonary injected with cells (5 × 10^6^) under anesthesia and then intranasally administered AAV5 particles (recommended normal intranasal dosage: 2 × 10^11^ vg, Genomeditech) 3 weeks later. The mice were euthanized and analyzed at 6 weeks after injection.

To generate PDX mouse models, 2 to 3 mm^3^ fresh LUAD tissues were subcutaneously implanted into athymic nude mice. After successful passage, the PDX mice were utilized for subsequent analysis. Tumor volumes were determined using the formula 0.5 × L × W^2^ (where L represents length and W represents width). The mice were humanely euthanized 36 days after passage.

All the tissue samples were recruited in Shanghai Chest Hospital from May 2013 to July 2023. The detailed information was summarized in Table S1. All the informed written consents were obtained. The studies including those for animals were approved by the institutional ethics committee of Shanghai Chest Hospital.

### Plasmids

METTL3 overexpression and knockout, YTHDC2^WT^, YTHDC2^Del-YTH^ overexpression, and exosome component 10 (EXOSC10) and YTHDC2 knockout plasmids were acquired from previous studies (15, 16). siRNA targeting CCL5, RSAD2, OAS2, IFI44, OAS1, MX2, IFIT3, IFIT2, CMPK2, XAF1, MAT1A, AANAT, SPEM2, LOC102723996, SMCO2, CD22, TAS1R3, DIPK1C, DIO3, KLK1 were purchased from GenePharma Co. Ltd. OAS1^WT^, OAS1^m6A mut^, IFIT1^WT^, IFIT1^m6A mut^, IFIT2^WT^, IFIT3^WT^, OAS2^WT^, OAS3^WT^, CCL5, RSAD2, MAT1A, AANAT, TAS1R3, METTL14 overexpression plasmids and shRNAs targeting YTHDC2 were purchased from Zorin. sgRNA sequences targeting YTHDC2, CCL5, RSAD2, OAS3, OAS2, OAS1, IFIT3, IFIT2, IFIT1, IFIT5, MAT1A, AANAT, TAS1R3, RNAseL, METTL3, XRN2, and RAB5A were listed in Table S2. IFIT1^D34A^, IFIT1^R38A^, IFIT1^Y157A^, IFIT1^R187A^, IFIT1^R255A^, IFIT1^R38A + R187A + R255A^, IFIT2^R41A + R184A + R258A^, IFTI3^R37A + R253A^, OAS1^Del-NT^, OAS2^Del-NT^, OAS3^Del-NT^, IFIT1^Del-TPR12^, IFIT3 ^Del-TPR12^, and IFIT5^Del-TPR12^ were constructed using overlapped PCR and the primers were listed in Table S2. The probes used for RNA-pulldown were also listed.

### IF, immunoblotting, and immunohistochemistry

For IF, we utilized Paul Karl Horan-67 (PKH67) (Sigma, #PKH67 GL) or PKH26 (Sigma, #PKH26 Gl) as a marker to identify exosome packaged in H1975/H1650 cells. Initially, exosome derived from H1650 or H1975 cells were mixed with Diluent C and PKH67 dye for a 5-min incubation period. The exosome was then isolated through three sequential centrifugation steps, following the procedure outlined below. Subsequently, the isolated exosome was incubated with H1975 or H1650 cells for 48 h. Following the incubation period, cells were harvested, fixed, and blocked. Nuclei were counter-stained with DAPI. Finally, all images were captured using a confocal microscope from Leica.

Immunoblotting (IB) was performed according to the routine protocol, which is available elsewhere. "The primary antibodies used for IB are as follows: anti-YTHDC2 (Abcam, #ab176846), anti-METTL3 (Abcam, #ab195352), anti-METTL14 (Abcam, #ab220030), anti-HA (Abcam, #ab1424 and #ab9110), anti-GAPDH (Abcam, #ab181602 and #ab8245), anti-CD63 (Abcam, #ab271286), anti-TSG101 (Abcam, #ab125011), anti-ALIX (Abcam, #ab275377), anti-CD9 (Abcam, #ab92726), anti-calnexin (Abcam, #ab133615), anti-RAB5A (Abcam, #ab66746), anti-RAB7 (Abcam, #ab137029), anti-RAB11A (Abcam, #ab128913), anti-IFIT1 (Abcam, #ab305301 and #ab118062), anti-IFIT3 (Abcam, #ab118045 and #236243), anti-FLAG (Abcam, #ab125243), anti-OAS1 (Abcam, #ab272492 and Sigma, MA5-43642), anti-OAS2 (Abcam, #ab195968 and #ab197655), anti-OAS3 (Abcam, #ab154270 and #ab188111), anti-IFIT2 (Abcam, #ab305231 and #ab168407), anti-IFIT5 (Abcam, #ab220954), anti-Myc (Cell Signaling Technology, #2276 and #2278), anti-CCL5 (Abcam, #ab307712), anti-RSAD2 (Abcam, #ab208286), anti-MAT1A (Abcam, #ab129176), anti-AANAT (Abcam, #ab3439), anti-TAS1R3 (Abcam, #ab229015), anti-LC3 (Abcam, #ab192890)." YTHDC2 protein level was also measured using ELISA kits from Lichen Biotechnology Co., LTD. The uncropped blots were displayed as Supplementary Materials.

For immunohistochemistry (IHC), the paraffin sections were hydrated on the first day, then cooked, washed with water, immersed in PBS solution, and coated with blocking endogenous peroxidase antibody, followed by DAB reaction detection, and finally counterstained sections. The IHC score was calculated by multiplying the staining intensity grade (0, 1, 2, or 3 for negative, weakly positive, moderately positive, and strongly positive, respectively) by the positivity score (0, 1, 2, 3, or 4 for 5%, 6–25%, 26–50%, 51–75%, and ≥76% of the positivity area, respectively). Antibodies used for IHC were as follows: anti-YTHDC2 (Abcam, #ab176846), anti-OAS1 (Abcam, #ab272492), anti-IFIT2 (Abcam, #ab305231), and anti-RAB5A (Abcam, #ab66746).

### Co-immunoprecipitation

For co-IP, cells were washed with PBS and lysed in Western/IP lysis buffer (Beyotime). Protein lysates were then incubated with 3 μg of the indicated antibodies and protein A/G beads (Invitrogen) overnight at 4 °C. After the immunoprecipitation reaction, the magnetic beads were washed with lysis buffer, finally added to SDS loading buffer, and subjected to IB analysis. The antibodies used were as follows: anti-Myc (CST, #2278), anti-OAS1 (Abcam, #ab272492), anti-OAS2 (Abcam, #ab197655), anti-OAS3 (Abcam, #ab154270), anti-IFIT1 (Abcam, #ab305301), anti-IFIT2 (Abcam, #ab305231), anti-IFIT3 (Abcam, #ab236243).

### RNA-immunoprecipitation

In the RIP assays, a Magna RIP Kit from Merck Millipore was cell lysates were subjected to overnight incubation at 4 °C with magnetic beads that were preloaded with either 5 μg of anti-IFIT1 (Abcam, #ab305301), anti-IFIT2 (Abcam, #ab305231), anti-IFIT3 (Abcam, #ab236243), anti-HA (Abcam, #ab1424), or anti-m^6^A antibodies (Abcam, #ab208577) or control IgG (CST, #3900). Following proteinase K digestion, the remaining RNA was extracted using TRIzol regent and subsequently analyzed through qPCR.

### Measurement of cell viability, caspase 3/7 activity, 4-HNE level, and anchorage-independent colony formation

Cell viability was detected using a CCK8 kit (Beyotime). Caspase 3/7 activity was determined using Caspase 3/7 Glo luciferase reagent (Promega). 4-HNE level was measured using kits from Abcam. Regarding the anchorage-independent colony formation assay, LUAD cells were plated in a six-well plate containing 0.3% agarose at a density of 6 × 10^3^ cells per well. After a 2-week incubation period, the colony numbers were evaluated using a microscope.

### Measurement of dual-luciferase activity

Luciferase activities were assessed by employing a dual-luciferase kit (Promega) in accordance with the provided instructions. Cells were cotransfected with Firefly reporters (primer used were listed in Table S2) and Renilla plasmids using Lipofectamine 2000 transfection reagent (Invitrogen). Following a 48-h incubation period, the cells were collected and lysed using the passive lysis buffer included in the kit. The fluorescence intensity of luciferase reporters was subsequently evaluated and normalized against the Renilla luciferase activity.

### Particle analysis

The absolute concentration and high-resolution size distribution of particle was determined by Flow NanoAnalyzer (NanoFCM).

### Dot-blot

For dot blot, the total RNA was extracted, the concentration was measured, and the total RNA was drawn on the nylon membrane as a 1 cm × 1 cm small square. The amount of RNA was calculated, and then the sample was loaded on the nylon membrane and irradiated under ultraviolet light for 10 min. After blocking in blocking solution for 1 hour, m^6^A primary antibody was prepared and incubated at 4 °C overnight. After washing with PBST, secondary antibodies were prepared and incubated at room temperature for 1 h. The exposure solution was prepared and then exposed.

### RNA pull-down

For RNA pull-down assays, RNA was labeled with a biotin probe, cellular proteins were lysed, and then subjected to precipitation of RNA, magnetic bead pretreatment, and IB or proteomic analysis.

### Photoactivatable ribonucleoside-enhanced crosslinking and immunoprecipitation

For PAR-CLIP, cells were incubated with (4-thiuridine, 4-SU, 250 μmol/L, Sigma) for 16 h and then irradiated with 355 nm UV light for cross-linking. Subsequently, cells were lysed with NP40 lysis buffer on ice and centrifuged at 18,000×*g* for 15 min to collect the supernatant, followed by a waiting period of 2 hours with antibody. After three washes with IP wash buffer, the beads were resuspended and boiled at 95 °C for 10 min. To detect antibodies-bound RNA, RNA was recovered and subjected to qPCR analysis.

### Statistical analysis

Tests used to examine the differences between groups were student’s *t* test, one-way, two-way ANOVA, log rank test, and the Spearman rank-correlation analysis. A *p* < 0.05 was considered statistically significant.

## Data availability

The accession number for the RNA-seq data reported in this paper is GEO: GSE241622.

## Supporting information

This article contains supporting information.

## Conflict of interest

The authors declare that they have no conflicts of interest with the contents of this article.

## References

[1] Sung H., Ferlay J., Siegel R.L., Laversanne M., Soerjomataram I., Jemal A. (2021). Global cancer statistics 2020: GLOBOCAN estimates of incidence and mortality worldwide for 36 cancers in 185 countries. CA Cancer J. Clin..

[2] Xia C., Dong X., Li H., Cao M., Sun D., He S. (2022). Cancer statistics in China and United States, 2022: profiles, trends, and determinants. Chin. Med. J. (Engl).

[3] Luo G., Zhang Y., Etxeberria J., Arnold M., Cai X., Hao Y. (2023). Projections of lung cancer incidence by 2035 in 40 countries worldwide: population-based study. JMIR Public Health Surveill..

[4] Hirsch F.R., Scagliotti G.V., Mulshine J.L., Kwon R., Curran W.J., Wu Y.L. (2017). Lung cancer: current therapies and new targeted treatments. Lancet.

[5] Qiu H., Cao S., Xu R. (2021). Cancer incidence, mortality, and burden in China: a time-trend analysis and comparison with the United States and United Kingdom based on the global epidemiological data released in 2020. Cancer Commun. (Lond).

[6] Kim S.Y., Kim S.M., Lim S., Lee J.Y., Choi S.J., Yang S.D. (2021). Modeling clinical responses to targeted therapies by patient-derived organoids of advanced lung adenocarcinoma. Clin. Cancer Res..

[7] Ng K.W., Boumelha J., Enfield K.S.S., Almagro J., Cha H., Pich O. (2023). Antibodies against endogenous retroviruses promote lung cancer immunotherapy. Nature.

[8] Alam S.K., Zhang Y., Wang L., Zhu Z., Hernandez C.E., Zhou Y. (2022). DARPP-32 promotes ERBB3-mediated resistance to molecular targeted therapy in EGFR-mutated lung adenocarcinoma. Oncogene.

[9] Huo F.C., Zhu Z.M., Pei D.S. (2020). N(6) -methyladenosine (m(6)A) RNA modification in human cancer. Cell Prolif..

[10] He L., Li H., Wu A., Peng Y., Shu G., Yin G. (2019). Functions of N6-methyladenosine and its role in cancer. Mol. Cancer.

[11] Mao Y., Dong L., Liu X.M., Guo J., Ma H., Shen B. (2019). m(6)A in mRNA coding regions promotes translation *via* the RNA helicase-containing YTHDC2. Nat. Commun..

[12] Fu Y., Zhuang X. (2020). m(6)A-binding YTHDF proteins promote stress granule formation. Nat. Chem. Biol..

[13] Li L., Krasnykov K., Homolka D., Gos P., Mendel M., Fish R.J. (2022). The XRN1-regulated RNA helicase activity of YTHDC2 ensures mouse fertility independently of m(6)A recognition. Mol. Cell.

[14] Hsu P.J., Zhu Y., Ma H., Guo Y., Shi X., Liu Y. (2017). Ythdc2 is an N(6)-methyladenosine binding protein that regulates mammalian spermatogenesis. Cell Res..

[15] Ma L., Zhang X., Yu K., Xu X., Chen T., Shi Y. (2021). Targeting SLC3A2 subunit of system X(C)(-) is essential for m(6)A reader YTHDC2 to be an endogenous ferroptosis inducer in lung adenocarcinoma. Free Radic. Biol. Med..

[16] Ma L., Chen T., Zhang X., Miao Y., Tian X., Yu K. (2021). The m(6)A reader YTHDC2 inhibits lung adenocarcinoma tumorigenesis by suppressing SLC7A11-dependent antioxidant function. Redox Biol..

[17] Zhao B.S., Roundtree I.A., He C. (2017). Post-transcriptional gene regulation by mRNA modifications. Nat. Rev. Mol. Cell Biol..

[18] Xu Z., Jia K., Wang H., Gao F., Zhao S., Li F. (2021). METTL14-regulated PI3K/Akt signaling pathway *via* PTEN affects HDAC5-mediated epithelial-mesenchymal transition of renal tubular cells in diabetic kidney disease. Cell Death Dis..

[19] Chen X., Lu T., Cai Y., Han Y., Ding M., Chu Y. (2023). KIAA1429-mediated m6A modification of CHST11 promotes progression of diffuse large B-cell lymphoma by regulating Hippo-YAP pathway. Cell Mol. Biol. Lett..

[20] Wang J., Yu H., Dong W., Zhang C., Hu M., Ma W. (2023). N6-Methyladenosine-Mediated up-regulation of FZD10 regulates liver cancer stem cells' properties and lenvatinib resistance through WNT/β-Catenin and Hippo signaling pathways. Gastroenterology.

[21] Shen C., Xuan B., Yan T., Ma Y., Xu P., Tian X. (2020). m(6)A-dependent glycolysis enhances colorectal cancer progression. Mol. Cancer.

[22] Chen Z., Wu L., Zhou J., Lin X., Peng Y., Ge L. (2020). N6-methyladenosine-induced ERRγ triggers chemoresistance of cancer cells through upregulation of ABCB1 and metabolic reprogramming. Theranostics.

[23] Zhu S., Wang J.Z., Chen D., He Y.T., Meng N., Chen M. (2020). An oncopeptide regulates m(6)A recognition by the m(6)A reader IGF2BP1 and tumorigenesis. Nat. Commun..

[24] Liu X., Gonzalez G., Dai X., Miao W., Yuan J., Huang M. (2020). Adenylate kinase 4 modulates the resistance of breast cancer cells to tamoxifen through an m(6)a-based epitranscriptomic mechanism. Mol. Ther..

[25] Kalluri R., LeBleu V.S. (2020). The biology, function, and biomedical applications of exosomes. Science.

[26] Mashouri L., Yousefi H., Aref A.R., Ahadi A.M., Molaei F., Alahari S.K. (2019). Exosomes: composition, biogenesis, and mechanisms in cancer metastasis and drug resistance. Mol. Cancer.

[27] Wortzel I., Dror S., Kenific C.M., Lyden D. (2019). Exosome-mediated metastasis: communication from a distance. Dev. Cell.

[28] An Y., Duan H. (2022). The role of m6A RNA methylation in cancer metabolism. Mol. Cancer.

[29] Wang J., Tan L., Jia B., Yu X., Yao R., N O.U. (2021). Downregulation of m(6)A reader YTHDC2 promotes the proliferation and migration of malignant lung cells *via* CYLD/NF-κB pathway. Int. J. Biol. Sci..

[30] Zhang X., Xu Y., Ma L., Yu K., Niu Y., Xu X. (2022). Essential roles of exosome and circRNA_101093 on ferroptosis desensitization in lung adenocarcinoma. Cancer Commun. (Lond).

[31] Yu W., Zhang C., Wang Y., Tian X., Miao Y., Meng F. (2023). YAP 5-methylcytosine modification increases its mRNA stability and promotes the transcription of exosome secretion-related genes in lung adenocarcinoma. Cancer Gene Ther..

[32] Xu X., Cui J., Wang H., Ma L., Zhang X., Guo W. (2022). IGF2BP3 is an essential N(6)-methyladenosine biotarget for suppressing ferroptosis in lung adenocarcinoma cells. Mater. Today Bio.

[33] Huang H., Weng H., Sun W., Qin X., Shi H., Wu H. (2018). Recognition of RNA N(6)-methyladenosine by IGF2BP proteins enhances mRNA stability and translation. Nat. Cell Biol..

[34] Memet I., Doebele C., Sloan K.E., Bohnsack M.T. (2017). The G-patch protein NF-κB-repressing factor mediates the recruitment of the exonuclease XRN2 and activation of the RNA helicase DHX15 in human ribosome biogenesis. Nucleic Acids Res..

[35] Makino D.L., Schuch B., Stegmann E., Baumgärtner M., Basquin C., Conti E. (2015). RNA degradation paths in a 12-subunit nuclear exosome complex. Nature.

[36] Fleith R.C., Mears H.V., Leong X.Y., Sanford T.J., Emmott E., Graham S.C. (2018). IFIT3 and IFIT2/3 promote IFIT1-mediated translation inhibition by enhancing binding to non-self RNA. Nucleic Acids Res..

[37] Abbas Y.M., Laudenbach B.T., Martínez-Montero S., Cencic R., Habjan M., Pichlmair A. (2017). Structure of human IFIT1 with capped RNA reveals adaptable mRNA binding and mechanisms for sensing N1 and N2 ribose 2'-O methylations. Proc. Natl. Acad. Sci. U. S. A..

[38] Schwartz S.L., Dey D., Tanquary J., Bair C.R., Lowen A.C., Conn G.L. (2022). Role of helical structure and dynamics in oligoadenylate synthetase 1 (OAS1) mismatch tolerance and activation by short dsRNAs. Proc. Natl. Acad. Sci. U. S. A..

[39] Xie H., Yao J., Wang Y., Ni B. (2022). Exosome-transmitted circVMP1 facilitates the progression and cisplatin resistance of non-small cell lung cancer by targeting miR-524-5p-METTL3/SOX2 axis. Drug Deliv..

[40] Ou B., Liu Y., Gao Z., Xu J., Yan Y., Li Y. (2022). Senescent neutrophils-derived exosomal piRNA-17560 promotes chemoresistance and EMT of breast cancer *via* FTO-mediated m6A demethylation. Cell Death Dis..

[41] Wang W., Qiao S.C., Wu X.B., Sun B., Yang J.G., Li X. (2021). Circ_0008542 in osteoblast exosomes promotes osteoclast-induced bone resorption through m6A methylation. Cell Death Dis..

[42] Hu Z., Chen G., Zhao Y., Gao H., Li L., Yin Y. (2023). Exosome-derived circCCAR1 promotes CD8 + T-cell dysfunction and anti-PD1 resistance in hepatocellular carcinoma. Mol. Cancer.

[43] Liu T., Wei Q., Jin J., Luo Q., Liu Y., Yang Y. (2020). The m6A reader YTHDF1 promotes ovarian cancer progression *via* augmenting EIF3C translation. Nucleic Acids Res..

[44] Wang S., Gao S., Zeng Y., Zhu L., Mo Y., Wong C.C. (2022). N6-Methyladenosine reader YTHDF1 promotes ARHGEF2 translation and RhoA signaling in colorectal cancer. Gastroenterology.

[45] Li Y., Banerjee S., Wang Y., Goldstein S.A., Dong B., Gaughan C. (2016). Activation of RNase L is dependent on OAS3 expression during infection with diverse human viruses. Proc. Natl. Acad. Sci. U. S. A..

[46] Kumar P., Sweeney T.R., Skabkin M.A., Skabkina O.V., Hellen C.U., Pestova T.V. (2014). Inhibition of translation by IFIT family members is determined by their ability to interact selectively with the 5'-terminal regions of cap0-, cap1- and 5'ppp- mRNAs. Nucleic Acids Res..

[47] Abbas Y.M., Pichlmair A., Górna M.W., Superti-Furga G., Nagar B. (2013). Structural basis for viral 5'-PPP-RNA recognition by human IFIT proteins. Nature.

[48] Wang J., Tan L., Yu X., Cao X., Jia B., Chen R. (2022). lncRNA ZNRD1-AS1 promotes malignant lung cell proliferation, migration, and angiogenesis *via* the miR-942/TNS1 axis and is positively regulated by the m(6)A reader YTHDC2. Mol. Cancer.

[49] Tan J., Chen F., Wang J., Li J., Ouyang B., Li X. (2024). ALKBH5 promotes the development of lung adenocarcinoma by regulating the polarization of M2 macrophages through CDCA4. Gene.

