# Supplementary material for: Downregulation of the m6A reader YTHDC2 upregulates exosome content in lung adenocarcinoma via inhibiting IFIT and OAS family members
Source: J Biol Chem. 2024 Sep 18;300(10):107783. doi: 10.1016/j.jbc.2024.107783 (PMC11736008; doi:10.1016/j.jbc.2024.107783)
Supplement: Supplementary Figure [file mmc1.pdf]

Figure S1

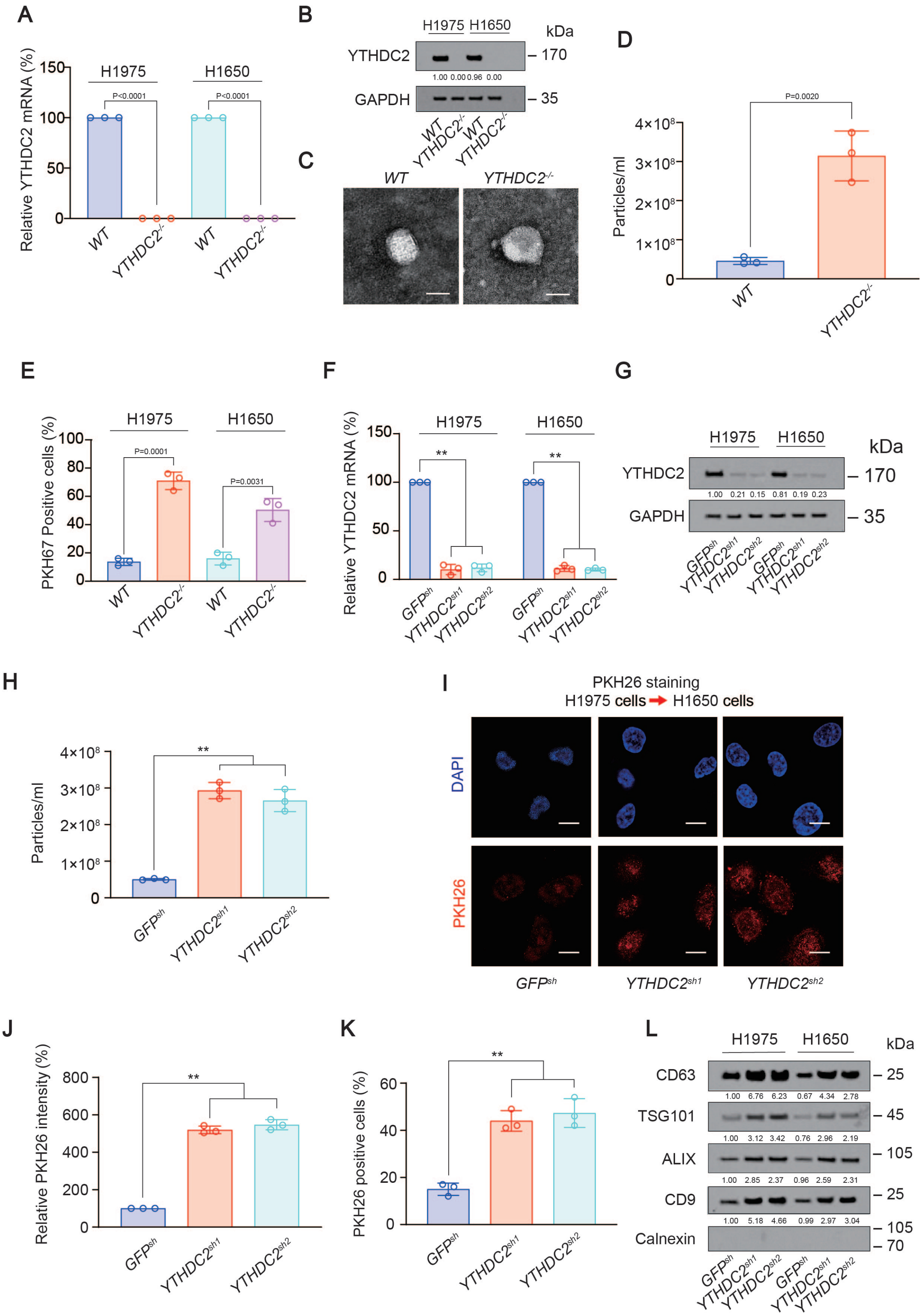

A
H1975
H1650
Relative YTHDC2 mRNA (%)
P<0.0001
WT
YTHDC2-/-
B
YTHDC2
GAPDH
kDa
170
35
1.00 0.00 0.96 0.00
C
D
Particles/ml
P=0.0020
E
PKH67 Positive cells (%)
P=0.0001
P=0.0031
F
Relative YTHDC2 mRNA (%)
**
GFPsh
YTHDC2sh1
YTHDC2sh2
G
1.00 0.21 0.15 0.81 0.19 0.23
H
I
PKH26 staining
H1975 cells → H1650 cells
DAPI
PKH26
J
Relative PKH26 intensity (%)
K
PKH26 positive cells (%)
L
CD63
TSG101
ALIX
CD9
Calnexin
1.00 6.76 6.23 0.67 4.34 2.78
1.00 3.12 3.42 0.76 2.96 2.19
1.00 2.85 2.37 0.96 2.59 2.31
1.00 5.18 4.66 0.99 2.97 3.04

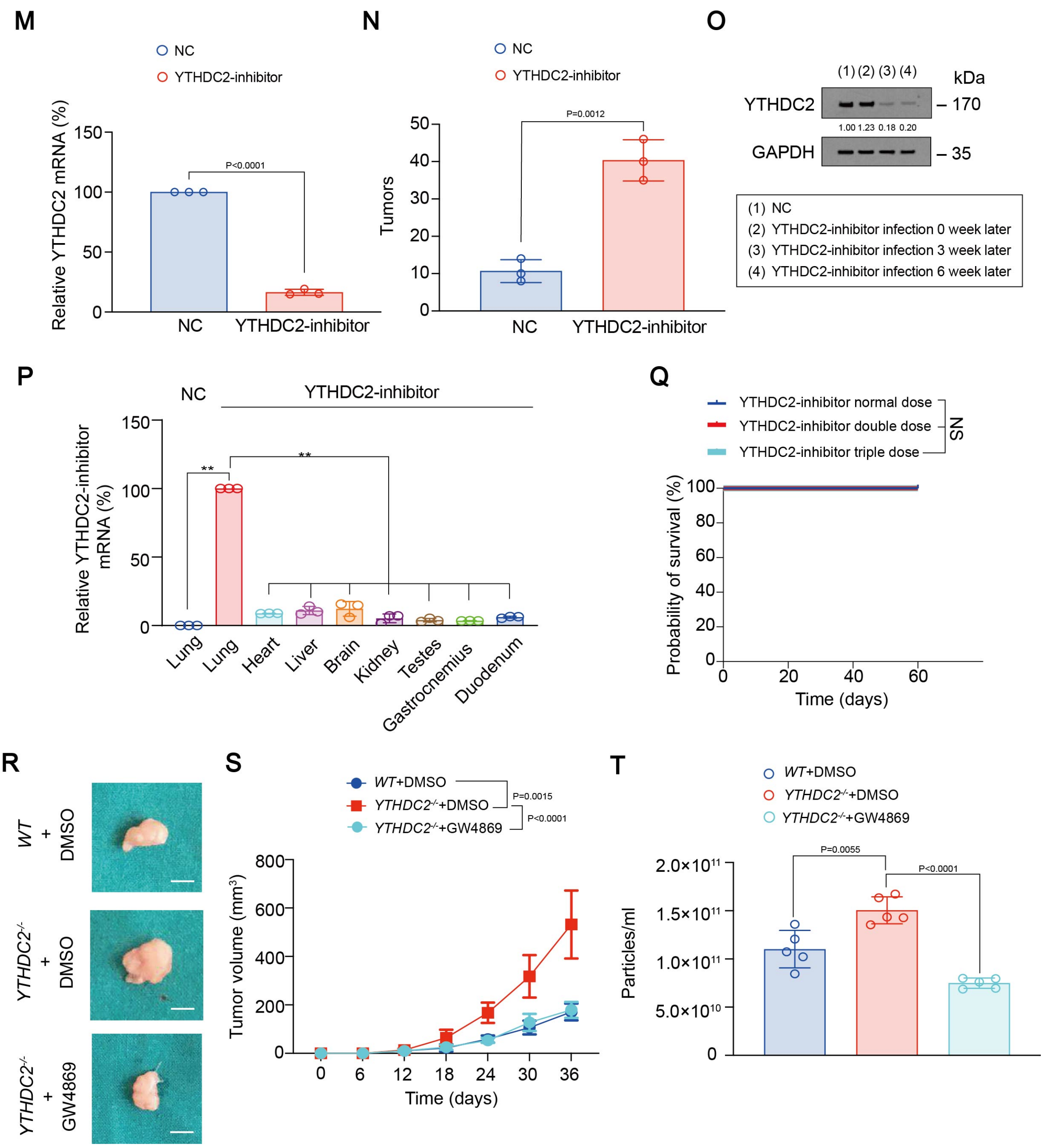

Figure S1. Supplementary to Figure 1. Knockout of YTHDC2 upregulated exosome concentration.

(A-B) YTHDC2 mRNA level (A) and protein level (B) was measured in *WT* or *YTHDC2$^{-/-}$* H1975 and H1650 cells. The level of proteins was normalized to that of GAPDH, and the normalized level of proteins in H1975 *WT* cells was arbitrarily set to 1.

(C) Representative TEM images of exosome in *WT* or *YTHDC2$^{-/-}$* H1975 cells. Scale bar, 50 nm.

(D) Particle concentration in *WT* or *YTHDC2$^{-/-}$* H1975 cells.

(E) Percent of PKH67 positive cells in Figure 1C.

(F-H) YTHDC2 mRNA level (F) and protein level (G), and particle concentration (H) in GFP^{sh} or $YTHDC2^{sh}$ infected H1975 and H1650 cells. The level of proteins was normalized to that of GAPDH, and the normalized level of proteins in GFP^{sh} infected H1975 cells was arbitrarily set to 1 (G).

(I-K) Transfer of exosome between GFP^{sh} or $YTHDC2^{sh}$ infected H1975 and H1650 cells. The exosome from was marked by PKH26 (green) and incubated with H1975 and H1650 cells. Afterward, IF was performed for the detection of exosome. Scale bar, 20 μm (I). The relative PKH26 intensity was measured using ImageJ (J). Percent of PKH26 positive cells was also graphed (K).

(L) CD63, TSG101, ALIX, CD9 and Calnexin was measured in exosome extracted from H1975 and H1650 cells. The level of proteins was normalized to that of GAPDH, and the normalized level of proteins in GFP^{sh} infected H1975 cells was arbitrarily set to 1. The number of cells in GFP^{sh} or $YTHDC2^{sh}$ samples was normalized to the same amount.

(M-N) YTHDC2 mRNA level (M) and tumors per lung (N) for the mouse models as described in Figure 1G.

(O) YTHDC2 protein level in H1975 cells injected into the lungs of nude mice at indicated times. The level of YTHDC2 proteins was normalized to that of GAPDH, and the normalized level of YTHDC2 proteins in NC sample was arbitrarily set to 1.

(P) Distribution content of YTHDC2 inhibitor in lung, heart, liver, brain, kidney, testes, gastrocnemius and duodenum as measured by qPCR.

(Q) Survival of mice after treatment with different doses of YTHDC2-inhibitor.

(R-T) Represented images (R), tumor volume (S) and particle concentration (T) for xenograft tumor formed by *WT* or $YTHDC2^{-/-}$ H1975 cells with or without GW4869 treatment. Scale bar, 5 mm.

The data are shown as the mean ± SD from three or five biological replicates. $^{**}P < 0.01$ indicates statistical significance. NS, non-significance. Data in Panel

A, D-E, M-N were analyzed by a student’s t-test. Data in Panel F, H, J-K, P, T were analyzed by a one-way ANOVA test. Data in Panel Q were analyzed by a log-rank test. Data in Panel S were analyzed by a two-way ANOVA test.

Figure S2

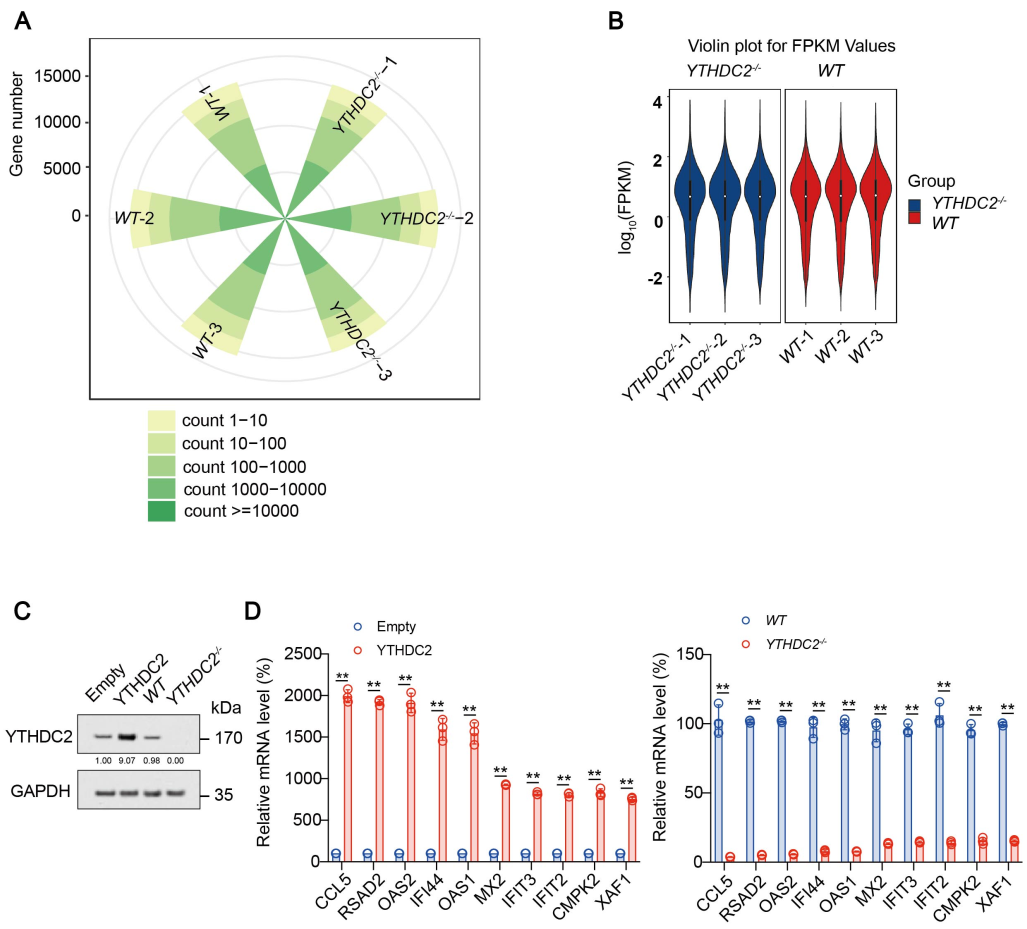

Figure S2. Supplementary to Figure 2. Identification of YTHDC2 downstream genes.

(A-B) Gene number (A) and FPKM values (B) for each group in RNA-seq.

(C) YTHDC2 overexpression and knockout validated by IB.

(D) Top ten YTHDC2 knockout downregulated mRNAs were validated by qPCR in YTHDC2 overexpressed or knockout H1975 cells.

The data are shown as the mean ± SD from three biological replicates. **P < 0.01 indicates statistical significance. Data in Panel D were analyzed by a student's t-test.

Figure S3

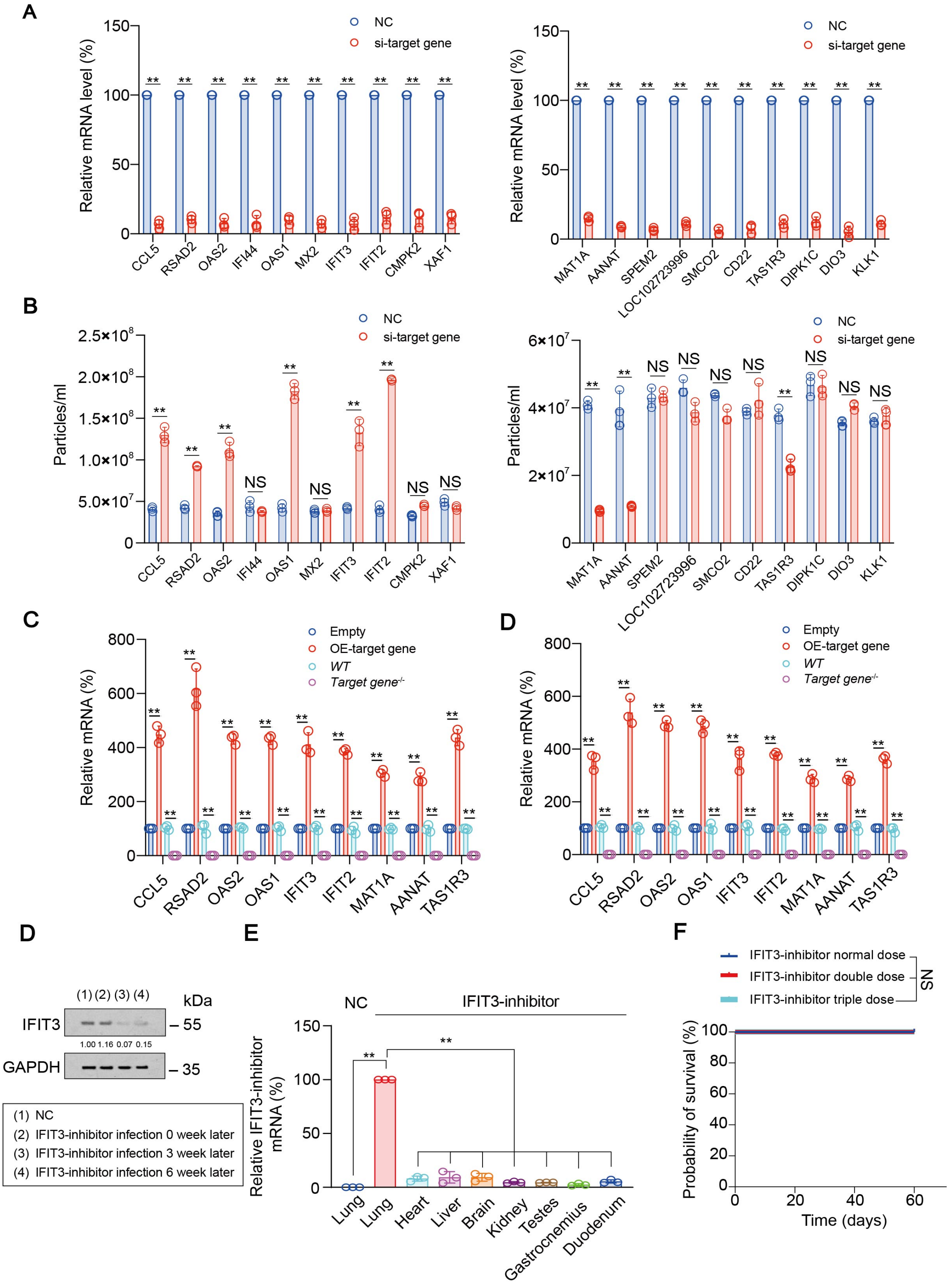

Figure S3. Supplementary to Figure 3. YTHDC2 downstream target genes could affect exosome concentration.

(A-B) Indicated mRNA levels measured with their siRNA treated (A). Particle concentration was also analyzed (B).

(C) Indicated mRNA levels measured with them overexpressed or knocked out.

(D) IFIT3 protein level in H1975 cells injected into the lungs of nude mice at indicated times. The level of IFIT3 proteins was normalized to that of GAPDH, and the normalized level of IFIT3 proteins in NC sample was arbitrarily set to 1.

(E) Distribution content of IFIT3 inhibitor in lung, heart, liver, brain, kidney, testes, gastrocnemius and duodenum as measured by qPCR.

(F) Survival of mice after treatment with different doses of IFIT3-inhibitor.

The data are shown as the mean ± SD from three biological replicates. $^{**}P < 0.01$ indicates statistical significance. NS, non-significance. Data in Panel A-C were analyzed by a student’s t-test. Data in Panel E were analyzed by a one-way ANOVA test. Data in Panel F were analyzed by a log-rank test.

Figure S4

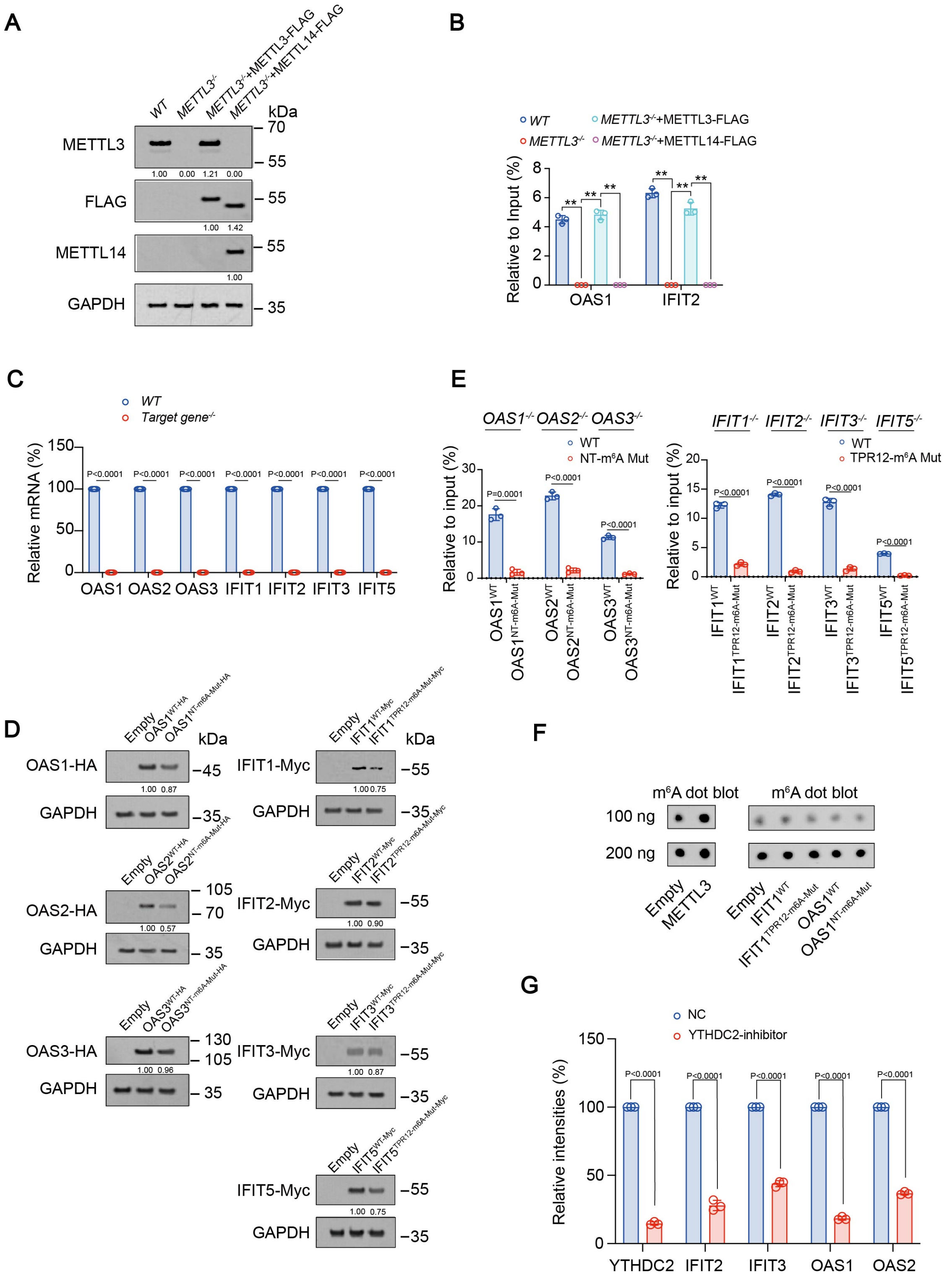

Figure S4. Supplementary to Figure 4. OAS and IFIT mRNAs were m^{6}A modified.

(A-B) METTL3 and METTL14 expression measured by IB in *WT* or *METTL3$^{-/-}$*

H1975 cells with or without METTL3/METTL14 FLAG overexpression (A). RNA-IP assay of RNA pulled down by anti-m^6A in these groups. OAS1 and IFIT2 mRNA levels in the pulled down products were verified by RT-qPCR (B).

(C) Indicated OAS and IFIT mRNA levels measured with them knocked out.

(D) OAS and IFIT protein expressions were measured by IB in H1975 cells with or without WT or m^6A-mut protein overexpression.

(E) RNA-IP assay of RNA pulled down by anti-m^6A in OAS or IFIT reconstituted H1975 cells. NT-m^6A mut indicated m^6A modification sites in OAS NT domain as shown in Figure 4B was mutated. TPR12-m^6A mut indicated m^6A modification sites in IFIT TPR12 domain as shown in Figure 4B was mutated. OAS and IFIT mRNA levels in the pulled down products were also verified by RT-qPCR.

(F) The m^6A level was analyzed by m^6A dot blot in H1975 cells with indicated plasmids overexpressed.

(G) IF intensities as measured by imageJ for Figure 4F.

The data are shown as the mean ± SD from three biological replicates. **$P < 0.01$ indicates statistical significance. NS, non-significance. Data in Panel B were analyzed by a one-way ANOVA test. Data in Panel C, E, G were analyzed by a student’s t-test.

Figure S5

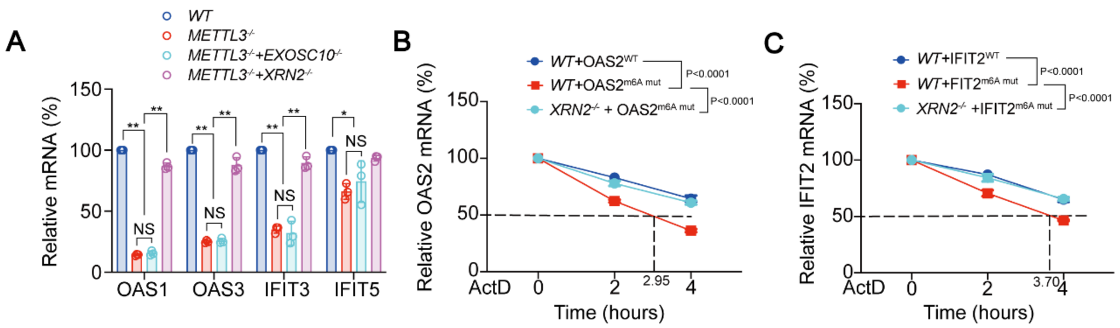

Figure S5. Supplementary to Figure 5. XRN2 was responsible for the stability of OAS and IFIT mRNAs.

(A) OAS1, OAS3, IFIT3 and IFIT5 mRNA levels were measured in *WT* or *METTL3*$^{-/-}$ H1650 cells with or without EXOSC10 or XRN2 knockout.

(B-C) OAS2 (B) and IFIT2 (C) mRNA stability was analyzed in^{-} H1650 cells with OAS2/IFIT2 WT or OAS2/IFIT2 m^{6}A mut overexpression with or without XRN2 knockout at indicated time after ActD treatment.

The data are shown as the mean ± SD from three biological replicates. *P < 0.05, **P < 0.01 indicates statistical significance. NS, non-significance. Data in Panel A were analyzed by a one-way ANOVA test. Data in Panel B-C were analyzed by a two-way ANOVA test.

Figure S6

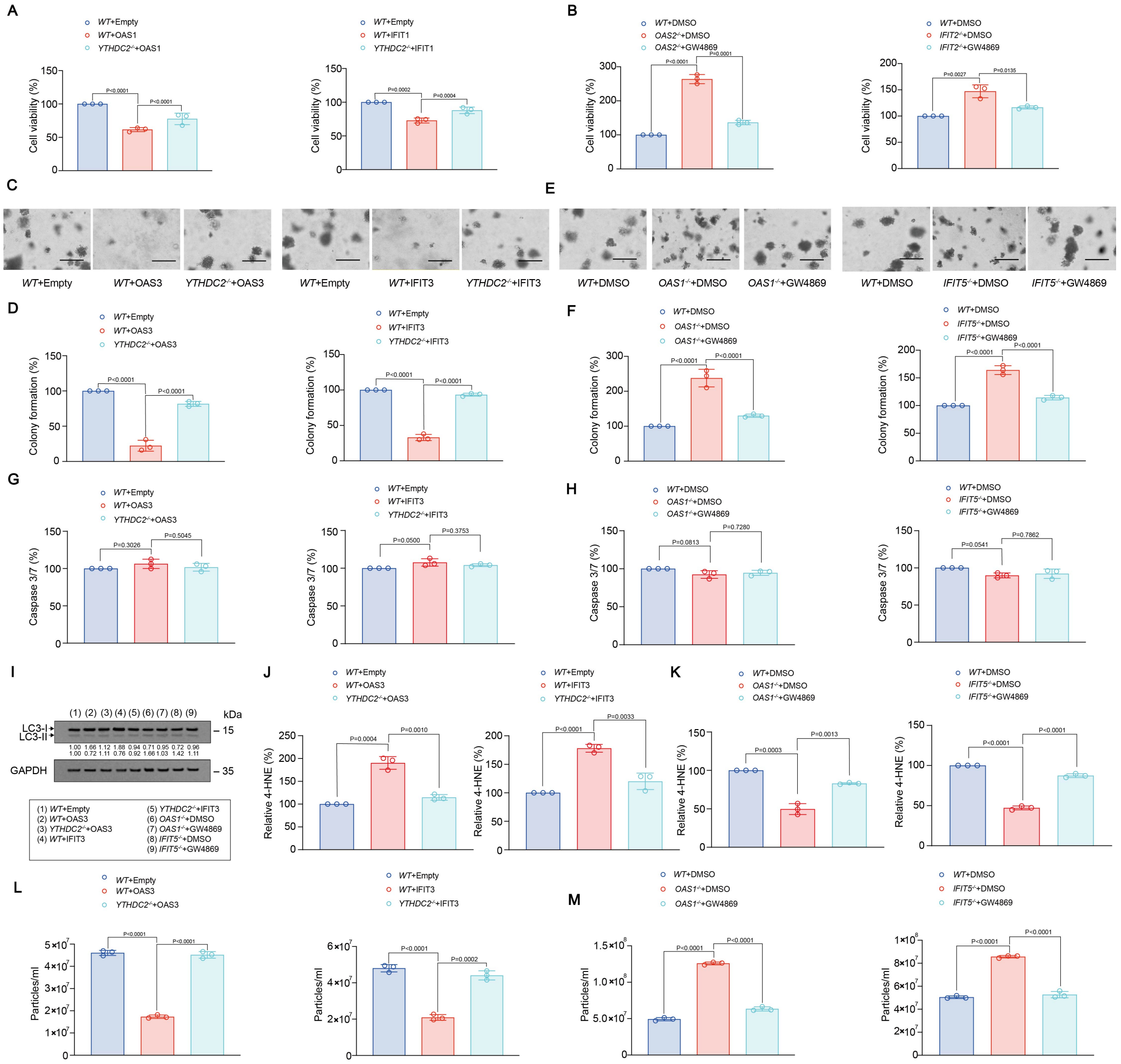

Figure S6. OAS and IFIT promoted malignant phenotype in LUAD cells.

(A) Cell viability was measured in control or OAS1/IFIT1 overexpressed H1975 cells with or without YTHDC2 knockout.

(B) Cell viability was measured in *WT* or *OAS2*$^{-/-}$/*IFIT2*$^{-/-}$ cells with or without GW4869 treatment.

(C-D) Representative images (C) and statistical results (D) for soft agar colony formation assay in control or OAS3/IFIT3 overexpressed H1975 cells with or without YTHDC2 knockout. Scale bar, 100 μm.

(E-F) Representative images (E) and statistical results (F) for soft agar colony formation assay in *WT* or *OAS1*$^{-/-}$/*IFIT5*$^{-/-}$ cells with or without GW4869 treatment. Scale bar, 100 μm.

(G) Caspase 3/7 activity was measured in control or OAS3/IFIT3

overexpressed H1975 cells with or without YTHDC2 knockout.

(H) Caspase 3/7 activity was measured in *WT* or *OAS1*$^{-/-}$/*IFIT5*$^{-/-}$ cells with or without GW4869 treatment.

(I) LC3 level was measured in control, OAS3/IFIT3 overexpressed H1975 cells with or without YTHDC2 knockout, or *OAS1*$^{-/-}$/*IFIT5*$^{-/-}$ H1975 cells with or without GW4869 treatment. The level of proteins was normalized to that of GAPDH, and the normalized level of proteins in control sample was arbitrarily set to 1.

(J) 4-HNE level was measured in control or OAS3/IFIT3 overexpressed H1975 cells with or without YTHDC2 knockout.

(K) 4-HNE level was measured in *WT* or *OAS1*$^{-/-}$/*IFIT5*$^{-/-}$ cells with or without GW4869 treatment.

(L) Particle concentration was measured in control or OAS3/IFIT3 overexpressed H1975 cells with or without YTHDC2 knockout.

(M) Particle concentration was measured in *WT* or *OAS1*$^{-/-}$/*IFIT5*$^{-/-}$ cells with or without GW4869 treatment.

The data are shown as the mean ± SD from three biological replicates. Data in Panel A, B, D, F-H, J-M were analyzed by a one-way ANOVA test.

Figure S7

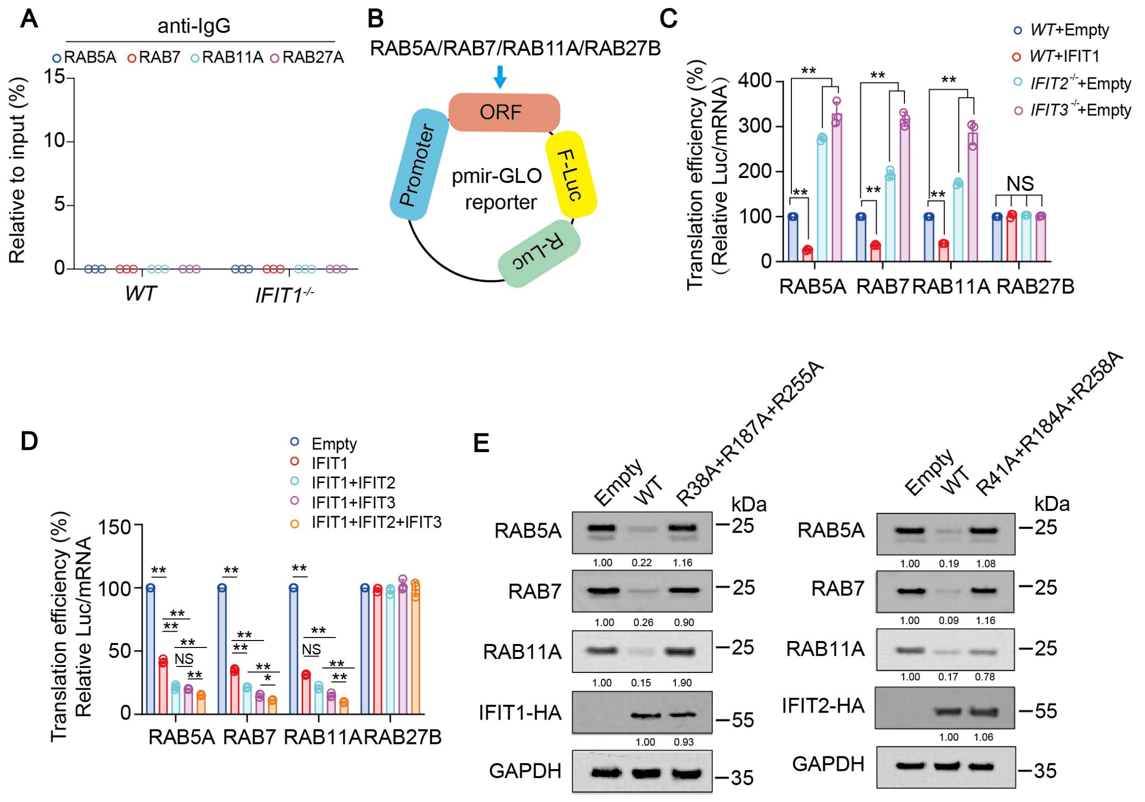

Figure S7. Supplementary to Figure 6. Mechanism that how IFIT triggered the degradation of target genes

(A) Parallel IgG control for Figure 6D.

(B) Schematic generation strategy for the pmir-Glo luciferase reporters containing RAB5A/RAB7/RAB11A/RAB27B CDS region.

(C) RAB5A, RAB7, RAB11A and RAB27B translation efficiency measured in H1975 cells with or without IFIT1 overexpression or IFIT2/IFIT3 knockout.

(D) RAB5A, RAB7, RAB11A and RAB27B translation efficiency measured in H1975 cells with or without IFIT1 overexpression combined with IFIT2 or IFIT3 overexpression.

(E) RAB5A, RAB7 and RAB11A expression measured by IB in H1975 cells with or without WT or indicated sites mutated IFIT1/IFIT2 overexpressed.

The data are shown as the mean ± SD from three biological replicates. *P < 0.05, **P < 0.01 indicates statistical significance. NS, non-significance. Data in Panel C, D were analyzed by a one-way ANOVA test.

Figure S8

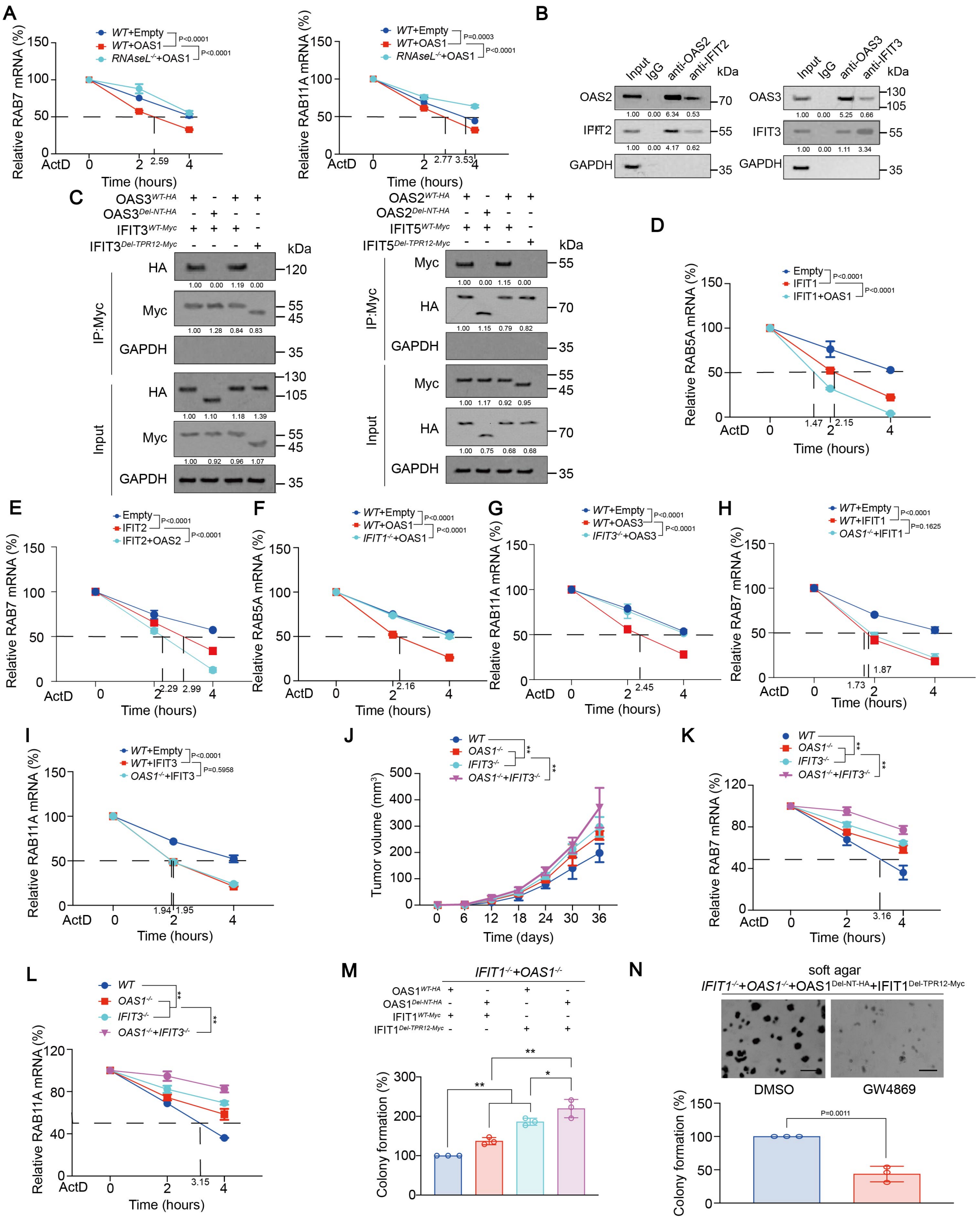

Figure S8. Supplementary to Figure 7. IFIT and OAS synergistically inhibited RAB5A, RAB7 and RAB11A.

(A) RAB7 and RAB11A mRNA stability was analyzed in control or OAS1 overexpression H1975 cells with or without RNAseL knockout at indicated time after ActD treatment.

(B) Co-IP experiments were performed using anti-OAS2/anti-IFIT2 or anti-

OAS3/anti-IFIT3 antibodies in H1975 cells. IgG-immunoprecipitated used samples were analyzed in parallel. Co-immunoprecipitated proteins were measured. The level of proteins in Input sample was arbitrarily set to 1.

(C) Co-IP experiments were performed using anti-Myc antibodies in H1975 cells with or without indicated WT or mutant OAS/IFIT overexpression. The level of proteins in OAS2/3$^{WT\text{-}HA}$ and IFIT3/5$^{WT\text{-}Myc}$ overexpression sample was arbitrarily set to 1.

(D-I) RAB5A, RAB7 or RAB11A mRNA stability was analyzed in H1975 cells with or without indicated plasmids overexpression or knockout at indicated time after ActD treatment.

(J-L) Tumor volume (J), RAB7 (K) and RAB11A (L) stability for xenograft tumor formed by *WT*, *OAS1*$^{-/-}$, *IFIT3*$^{-/-}$, *OAS1*$^{-/-}$+*IFIT3*$^{-/-}$ H1975 cells.

(M) Statistical results for Figure 7J.

(N) Soft agar colony formation assay in IFIT1 and OAS1 reconstituted H1650 cells with OAS1$^{WT\text{-}HA}$, OAS1$^{Del\text{-}NT\text{-}HA}$, IFIT1$^{WT\text{-}Myc}$ or IFIT1$^{Del\text{-}TPR12\text{-}Myc}$ overexpression treated with or without GW4869. Scale bar, 100 μm.

The data are shown as the mean ± SD from three biological replicates. $^*P < 0.05$, $^{**}P < 0.01$ indicates statistical significance. Data in Panel A, D-L were analyzed by a two-way ANOVA test. Data in Panel M were analyzed by a one-way ANOVA test. Data in Panel N were analyzed by a student's t test.

Figure S9

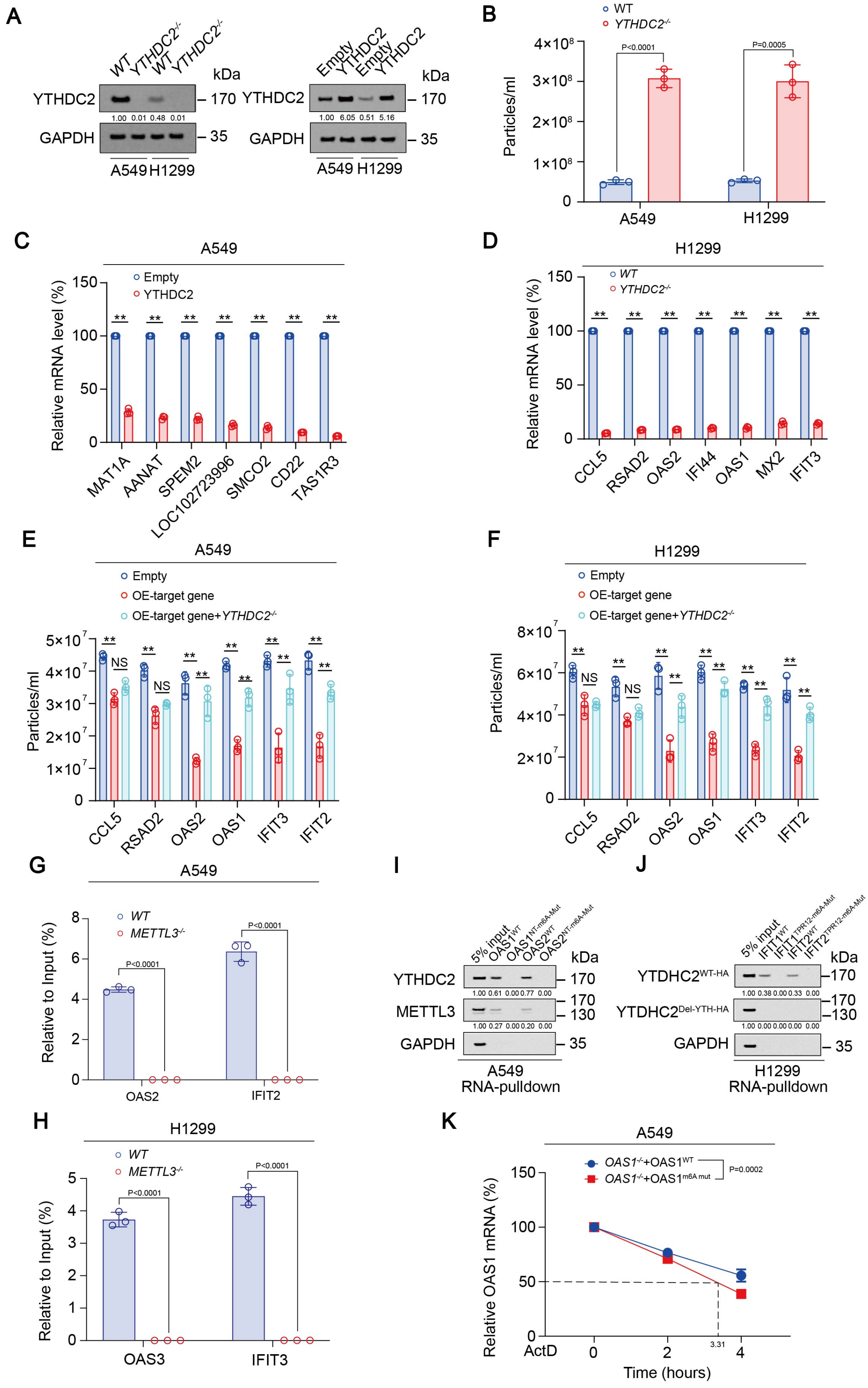
A
YTHDC2
GAPDH
kDa
170
35
A549 H1299
B
WT
YTHDC2-/-
Particles/ml
P<0.0001
P=0.0005
A549
H1299
C
A549
Empty
YTHDC2
Relative mRNA level (%)
MAT1A AANAT SPEM2 LOC102723996 SMCO2 CD22 TAS1R3
D
H1299
Relative mRNA level (%)
CCL5 RSAD2 OAS2 IFI44 OAS1 MX2 IFIT3
E
A549
Empty
OE-target gene
OE-target gene+YTHDC2-/-
Particles/ml
CCL5 RSAD2 OAS2 OAS1 IFIT3 IFIT2
F
H1299
G
A549
WT
METTL3-/-
Relative to Input (%)
OAS2
IFIT2
H
H1299
OAS3
IFIT3
I
YTHDC2
METTL3
GAPDH
A549
RNA-pulldown
J
YTDHC2WT-HA
YTDHC2Del-YTH-HA
GAPDH
H1299
RNA-pulldown
K
A549
OAS1-/-+OAS1WT
OAS1-/-+OAS1m6A mut
P=0.0002
Relative OAS1 mRNA (%)
ActD
Time (hours)
3.31

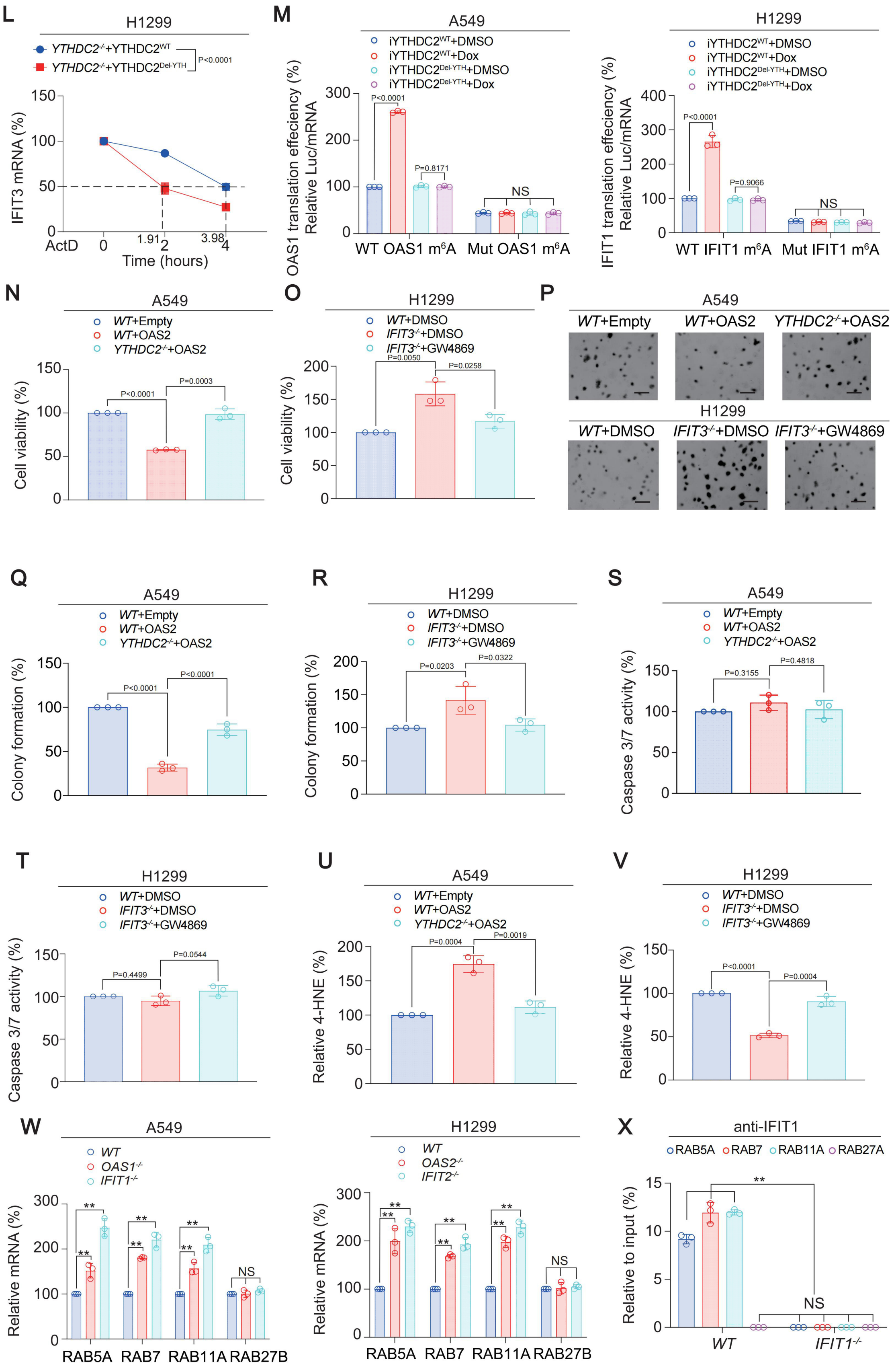
L
H1299
YTHDC2-/-+YTHDC2WT
YTHDC2-/-+YTHDC2Del-YTH
P<0.0001
IFIT3 mRNA (%)
ActD
Time (hours)
1.91
3.98
M
A549
iYTHDC2WT+DMSO
iYTHDC2WT+Dox
iYTHDC2Del-YTH+DMSO
iYTHDC2Del-YTH+Dox
OAS1 translation effeciency (%)
Relative Luc/mRNA
P<0.0001
P=0.8171
NS
WT OAS1 m6A
Mut OAS1 m6A
H1299
IFIT1 translation effeciency (%)
Relative Luc/mRNA
P<0.0001
P=0.9066
NS
WT IFIT1 m6A
Mut IFIT1 m6A
N
A549
WT+Empty
WT+OAS2
YTHDC2-/-+OAS2
Cell viability (%)
P<0.0001
P=0.0003
O
H1299
WT+DMSO
IFIT3-/-+DMSO
IFIT3-/-+GW4869
Cell viability (%)
P=0.0050
P=0.0258
P
A549
WT+Empty
WT+OAS2
YTHDC2-/-+OAS2
H1299
WT+DMSO
IFIT3-/-+DMSO
IFIT3-/-+GW4869
Q
A549
Colony formation (%)
P<0.0001
P<0.0001
R
H1299
Colony formation (%)
P=0.0203
P=0.0322
S
A549
Caspase 3/7 activity (%)
P=0.3155
P=0.4818
T
H1299
Caspase 3/7 activity (%)
P=0.4499
P=0.0544
U
A549
Relative 4-HNE (%)
P=0.0004
P=0.0019
V
H1299
Relative 4-HNE (%)
P<0.0001
P=0.0004
W
A549
WT
OAS1-/-
IFIT1-/-
Relative mRNA (%)
RAB5A
RAB7
RAB11A
RAB27B
NS
H1299
WT
OAS2-/-
IFIT2-/-
Relative mRNA (%)
RAB5A
RAB7
RAB11A
RAB27B
NS
X
anti-IFIT1
RAB5A
RAB7
RAB11A
RAB27A
Relative to input (%)
NS
WT
IFIT1-/-

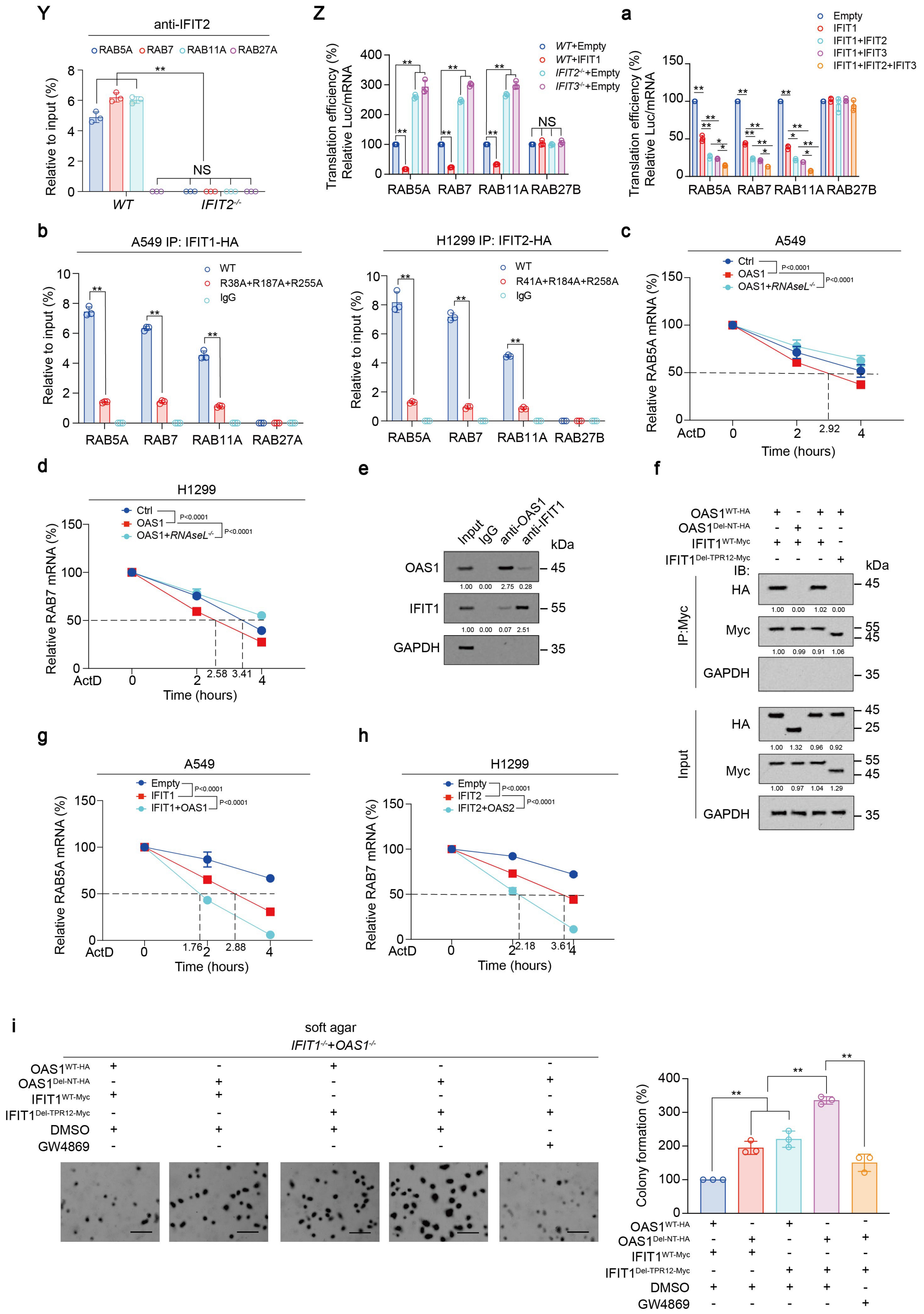

Figure S9. Mechanism validated in A549 and H1299 cells.

(A) YTHDC2 protein level in YTHDC2 overexpression or knockout A549 and H1299 cells as measured by IB. The level of proteins was normalized to that

of GAPDH, and the normalized level of proteins in control group was arbitrarily set to 1.

(B) Particle concentration in *WT* or *YTHDC2*$^{-/-}$ A549 and H1299 cells.

(C-D) mRNA level of indicated genes in A549 cells with YTHDC2 overexpression (C) or in H1299 cells with YTHDC2 knockout (D).

(E-F) Particle concentration was measured in A549 (E) or H1299 (F) cells with target gene overexpression with or without YTHDC2 knockout.

(G-H) RNA-IP assay of RNA pulled down by anti-m^{6}A in *WT* or *METTL3*$^{-/-}$ groups. OAS2 and IFIT2 mRNA levels in the A549 pulled down products (G) and OAS3 and IFIT3 mRNA levels in the H1299 pulled down products (H) were verified by RT-qPCR.

(I-J) RNA pull-down experiments in A549 (I) or H1299 (J) cells using WT or m^{6}A mutant OAS1/2 (I) or IFIT1/2 (J) probes, as indicated, and the interaction of YTHDC2 and METTL3 was revealed by IB. The level of proteins in 5% input sample was arbitrarily set to 1.

(K) OAS1 mRNA stability was analyzed in *OAS1*$^{-/-}$ A549 cells with OAS1 WT or OAS1 m^{6}A mut overexpression at indicated time after ActD treatment.

(L) IFIT3 mRNA stability was analyzed in *YTHDC2*$^{-/-}$ H1299 cells with YTHDC2^{WT} or YTHDC2$^{Del\text{-}YTH}$ overexpression at indicated time after ActD treatment.

(M) Luciferase activities from the pmir-Glo vector containing WT or m^{6}A site mutant OAS1 or IFIT1 CDS region were measured in A549 or H1299 cells with or without Dox-inducible YTHDC2^{WT} or YTHDC2$^{Del\text{-}YTH}$ overexpression with or without Dox treatment. Translation efficiency was calculated as the ratio of luciferase activity to mRNA content.

(N) Cell viability was measured in control or OAS2 overexpressed A549 cells with or without YTHDC2 knockout.

(O) Cell viability was measured in *WT* or *IFIT3*$^{-/-}$ H1299 cells with or without GW4869 treatment.

(P) Representative images for soft agar colony formation assay in control or OAS2 overexpressed A549 cells with or without YTHDC2 knockout, or in *WT* or *IFIT3*$^{-/-}$ H1299 cells with or without GW4869 treatment. Scale bar, 100 μm.

(Q-R) Statistical results for Panel P.

(S) Caspase 3/7 activity was measured in control or OAS2 overexpressed A549 cells with or without YTHDC2 knockout.

(T) Caspase 3/7 activity was measured in *WT* or *IFIT3*$^{-/-}$ H1299 cells with or without GW4869 treatment.

(U) 4-HNE level was measured in control or OAS2 overexpressed A549 cells with or without YTHDC2 knockout.

(V) 4-HNE level was measured in *WT* or *IFIT3*$^{-/-}$ H1299 cells with or without GW4869 treatment.

(W) RAB5A, RAB7, RAB11A and RAB27B mRNA level in A549 cells with or without OAS1 or IFIT1 knockout or H1299 cells with or without OAS2 or IFIT2 knockout.

(X-Y) RNA-IP assay of RNA pulled down by anti-IFIT1 (X) or anti-IFIT2 (Y) in A549 cells with or without IFIT1 or IFIT2 knockout. RAB5A, RAB7, RAB11A and RAB27A mRNA levels in the pulled down products were also verified by RT-qPCR.

(Z) RAB5A, RAB7, RAB11A and RAB27B translation efficiency measured in A549 cells with or without IFIT1 overexpression or IFIT2/IFIT3 knockout.

(a) RAB5A, RAB7, RAB11A and RAB27B translation efficiency measured in A549 cells with or without IFIT1 overexpression combined with IFIT2 or IFIT3 overexpression.

(b) RNA-IP assay of RNA pulled down by HA-tagged IFIT1 or IFIT2 (I) with indicated site mutated in A549 or H1299 cells. RAB5A, RAB7, RAB11A, RAB27A or RAB27B mRNA levels in the pulled down products were also verified by RT-qPCR.

(c-d) RAB5A (c) and RAB7 (d) mRNA stability was analyzed in A549 (c) or

H1299 (d) cells with OAS1 overexpression with or without RNAseL knockout at indicated time after ActD treatment.

(e) Co-IP experiments were performed using anti-OAS1 or anti-IFIT1 antibodies in A549 cells. IgG-immunoprecipitated used samples were analyzed in parallel. Co-immunoprecipitated OAS1 and IFIT1 expression was measured. The level of proteins in Input sample was arbitrarily set to 1.

(f) Co-IP experiments were performed using anti-Myc antibodies in A549 cells with or without $OAS1^{WT-HA}$, $OAS1^{Del-NT-HA}$, $IFIT1^{WT-Myc}$ or $IFIT1^{Del-TPR12-Myc}$ overexpression. The level of proteins in $OAS1^{WT-HA}$ and $IFIT1^{WT-Myc}$ overexpression sample was arbitrarily set to 1.

(g) RAB5A mRNA stability was analyzed in A549 cells with or without IFIT1 and OAS1 overexpression at indicated time after ActD treatment.

(h) RAB7 mRNA stability was analyzed in H1299 cells with or without IFIT2 and OAS2 overexpression at indicated time after ActD treatment.

(i) Representative images and statistical analysis for soft agar colony formation assay in IFIT1 and OAS1 reconstituted A549 cells with $OAS1^{WT-HA}$, $OAS1^{Del-NT-HA}$, $IFIT1^{WT-Myc}$ or $IFIT1^{Del-TPR12-Myc}$ overexpression treated with or without GW4869. Scale bar, 100 μm.

The data are shown as the mean ± SD from three biological replicates. *$P < 0.05$, **$P < 0.01$ indicates statistical significance. NS, non-significance. Data in Panel B-D, G, H, b were analyzed by a student's t test. Data in Panel E, F, M-O, Q-a, i were analyzed by a one-way ANOVA test. Data in Panel K, L, c, d, g, h were analyzed by a two-way ANOVA test.

Figure S10

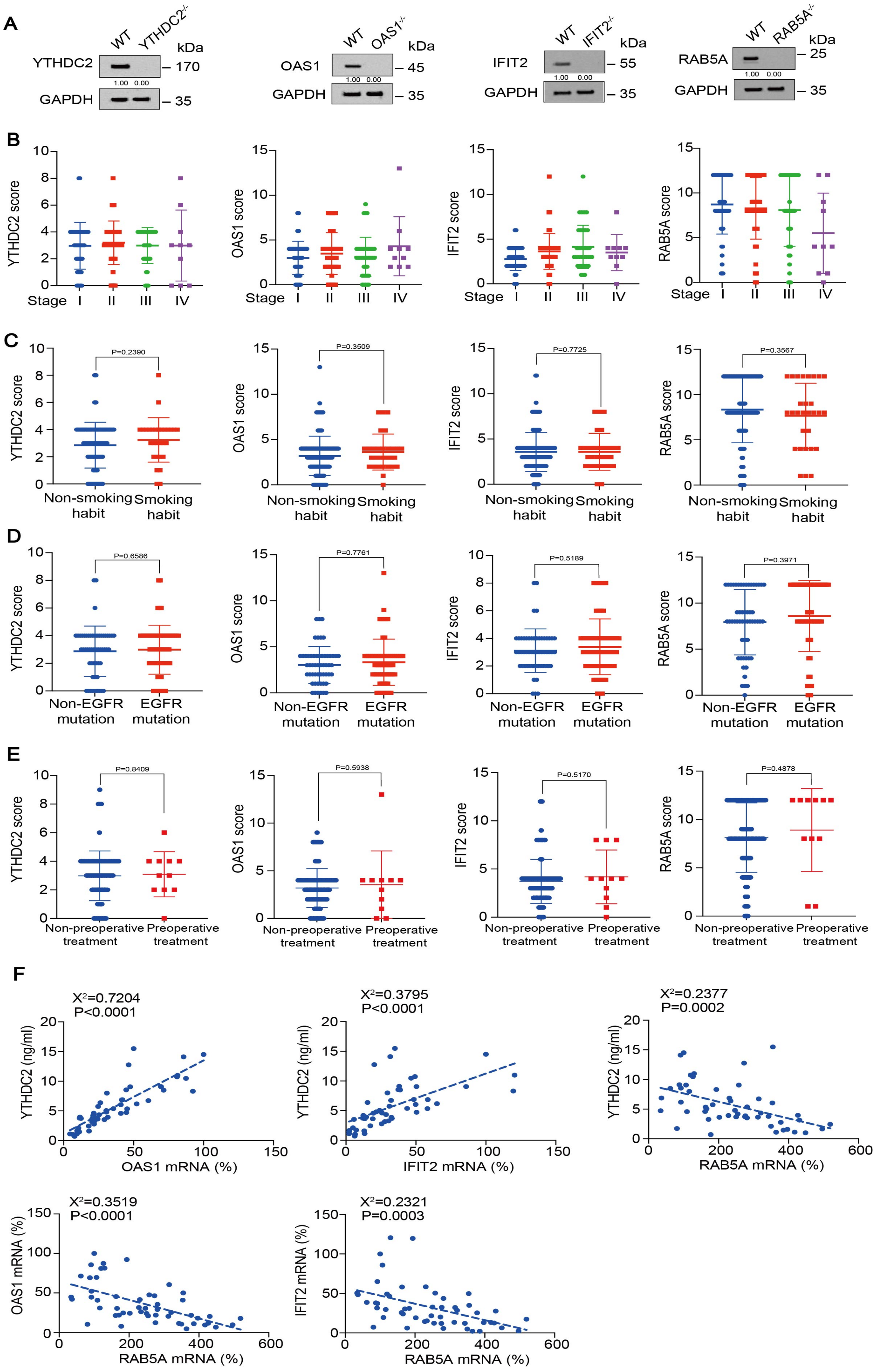

A
WT
YTHDC2-/-
kDa
YTHDC2
170
1.00 0.00
GAPDH
35
WT
OAS1-/-
OAS1
45
IFIT2-/-
IFIT2
55
RAB5A-/-
RAB5A
25
B
YTHDC2 score
OAS1 score
IFIT2 score
RAB5A score
Stage
I
II
III
IV
C
P=0.2390
P=0.3509
P=0.7725
P=0.3567
Non-smoking habit
Smoking habit
D
P=0.6586
P=0.7761
P=0.5189
P=0.3971
Non-EGFR mutation
EGFR mutation
E
P=0.8409
P=0.5938
P=0.5170
P=0.4878
Non-preoperative treatment
Preoperative treatment
F
X²=0.7204
P<0.0001
X²=0.3795
P<0.0001
X²=0.2377
P=0.0002
YTHDC2 (ng/ml)
OAS1 mRNA (%)
IFIT2 mRNA (%)
RAB5A mRNA (%)
X²=0.3519
P<0.0001
X²=0.2321
P=0.0003

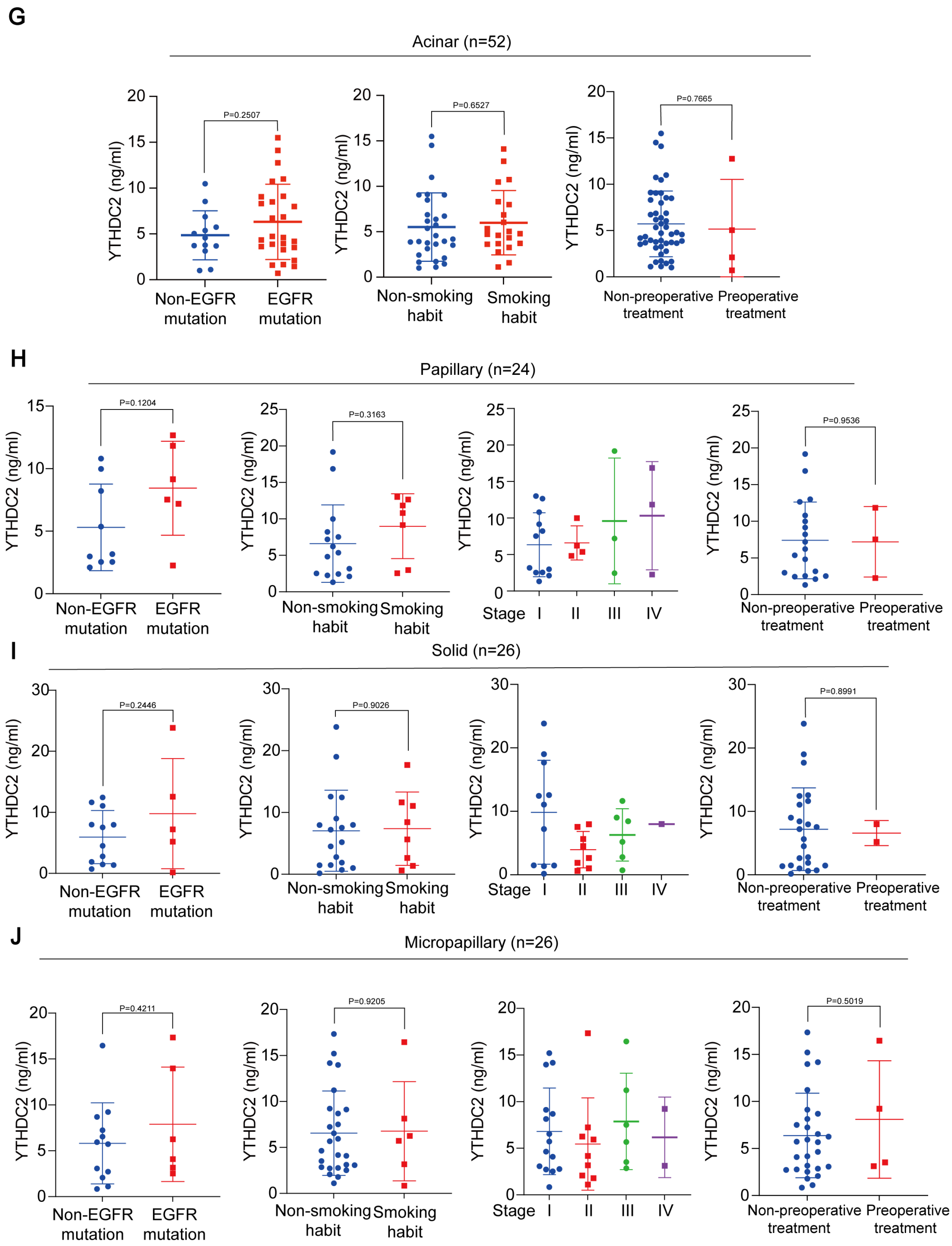

Figure S10. Supplementary to Figure 8. Clinical significance of YTHDC2, OAS1, IFIT2 and RAB5A in LUAD and its acinar subtype.

(A) Validation of anti-YTHDC2, anti-OAS1, anti-IFIT2, and anti-RAB5A for tissue microarray in *WT* and target protein knockout H1975 cells.

(B-E) The results of Figure 9B were further presented according to the tumor stage (B), smoking habit (C), EGFR mutation status (D) and whether receiving

preoperative treatment (including targeted therapy, immunotherapy, chemoradiotherapy and so on) (E).

(F) Correlation among YTHDC2, OAS1, IFIT2 and RAB5A in acinar subtype LUAD.

(G) The results of Figure 9C were further presented according to the smoking habit, EGFR mutation status, and whether receiving preoperative treatment.

(H-J) YTHDC2 protein level were measured in papillary (H), solid (I) and micropapillary (J) subtype LUAD and further presented according to the tumor stage, smoking habit, EGFR mutation status and whether receiving preoperative treatment.

Data in Panel C-E, G were analyzed by a student's t test. Data in Panel F were analyzed by a Spearman's rank correlation analysis. Data in Panel H-J were analyzed by a student's t test.
