# Supplementary material for: Downregulation of the m6A reader YTHDC2 upregulates exosome content in lung adenocarcinoma via inhibiting IFIT and OAS family members
Source: J Biol Chem. 2024 Sep 18;300(10):107783. doi: 10.1016/j.jbc.2024.107783 (PMC11736008; doi:10.1016/j.jbc.2024.107783)
Supplement: Supplementary Table 1 [file mmc2.docx]

**Supplementary Table 1. Basal information of patients.**

**LUAD patient for tissue microarray**

| LUAD patient (N=181) | | |
| --- | --- | --- |
| Age |  |  |
|  | ≥65 | 93 |
|  | <65 | 88 |
|  |  |  |
| Gender |  |  |
|  | Male | 95 |
|  | Female | 93 |
| Smoking |  |  |
|  | Yes | 29 |
|  | No | 132 |
|  | Not Reported | 20 |
|  |  |  |
| EGFR mutation | |  |
|  | Yes | 64 |
|  | No | 54 |
|  | Not Reported | 63 |

**Subtype LUAD patient for further analysis**

| Acinar LUAD patient (N=52) | | |
| --- | --- | --- |
| Age |  |  |
|  | ≥65 | 27 |
|  | <65 | 25 |
|  |  |  |
|  | Male | 30 |
|  | Female | 22 |
|  |  |  |
| Smoking |  |  |
|  | Yes | 20 |
|  | No | 29 |
|  | Not Reported | 3 |
|  |  |  |
| EGFR mutation | |  |
|  | Yes | 26 |
|  | No | 13 |
|  | Not Reported | 13 |

| Papillary LUAD patient (N=22) | | |
| --- | --- | --- |
| Age |  |  |
|  | ≥65 | 11 |
|  | <65 | 11 |
|  |  |  |
| Gender |  |  |
|  | Male | 12 |
|  | Female | 10 |

| Solid LUAD patient (N=26) | | |
| --- | --- | --- |
| Age |  |  |
|  | ≥65 | 14 |
|  | <65 | 12 |
|  |  |  |
| Gender |  |  |
|  | Male | 13 |
|  | Female | 13 |

| Micropapillary LUAD patient (N=32) | | |
| --- | --- | --- |
| Age |  |  |
|  | ≥65 | 18 |
|  | <65 | 14 |
|  |  |  |
| Gender |  |  |
|  | Male | 17 |
|  | Female | 15 |
