# Supplementary material for: Downregulation of the m6A reader YTHDC2 upregulates exosome content in lung adenocarcinoma via inhibiting IFIT and OAS family members
Source: J Biol Chem. 2024 Sep 18;300(10):107783. doi: 10.1016/j.jbc.2024.107783 (PMC11736008; doi:10.1016/j.jbc.2024.107783)
Supplement: Supplementary materials [file mmc4.docx]

**Supplementary Materials. Images for original blots.**

**Blots 1**

**
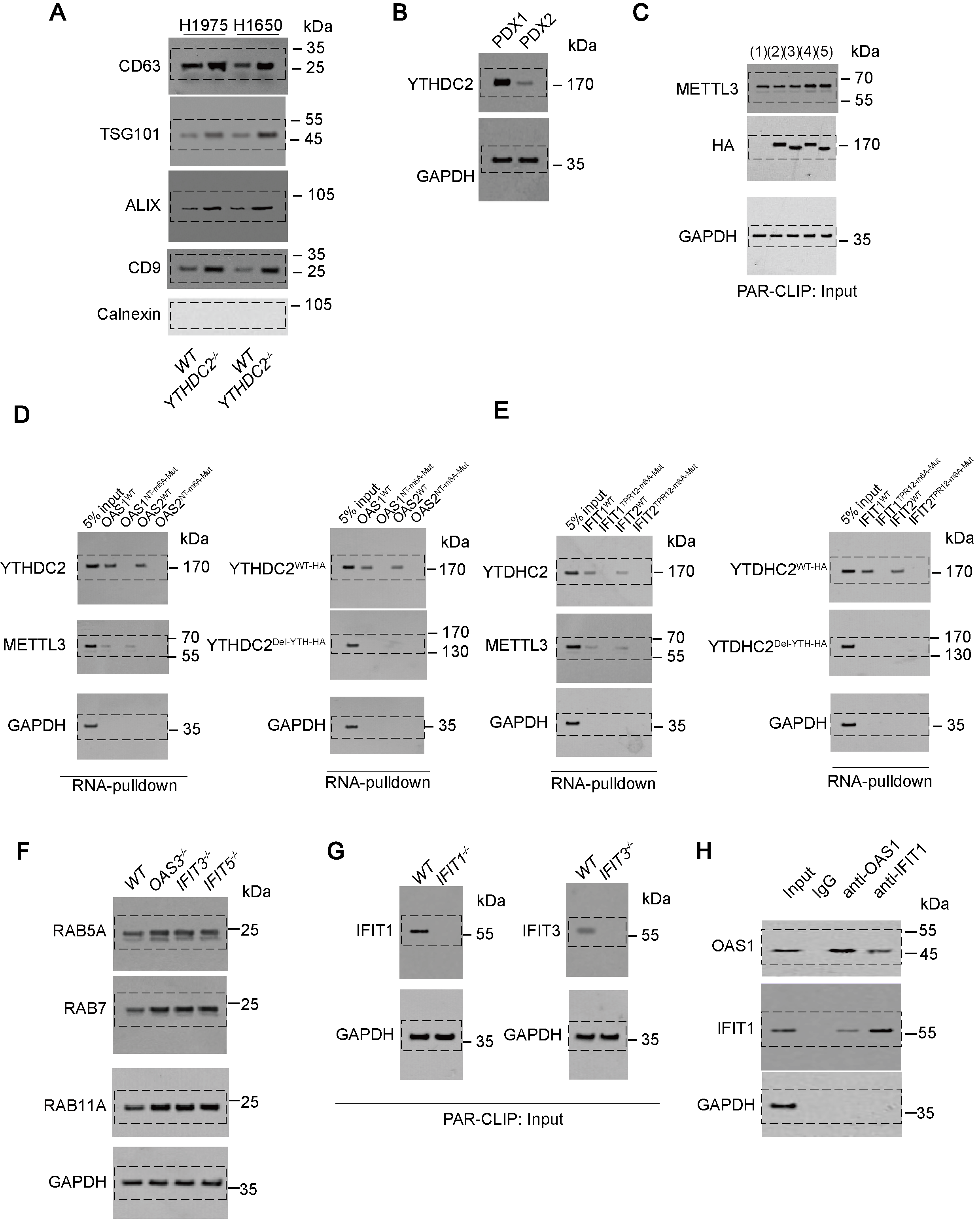
**

**
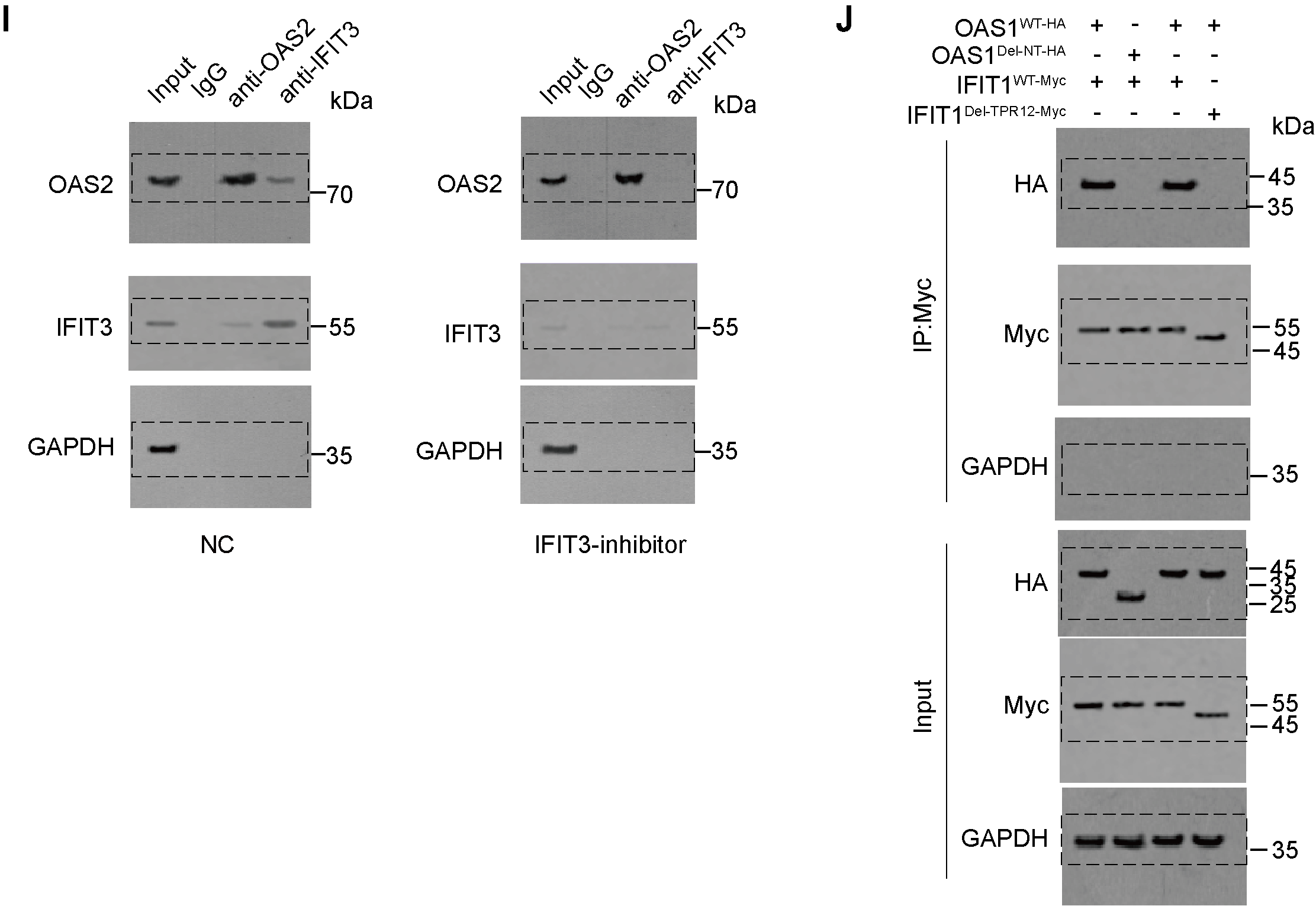
**

**Blots 1: Images for original blots 1**

(A) Original blots for Fig. 1E.

(B) Original blots for Fig. 1K.

(C) Original blots for Fig. 4C.

(D) Original blots for Fig. 4D.

(E) Original blots for Fig. 4E.

(F) Original blots for Fig. 6C.

(G) Original blots for Fig. 6E.

(H) Original blots for Fig. 7C.

(I) Original blots for Fig. 7D.

(J) Original blots for Fig. 7E.

**Blots 2**

**
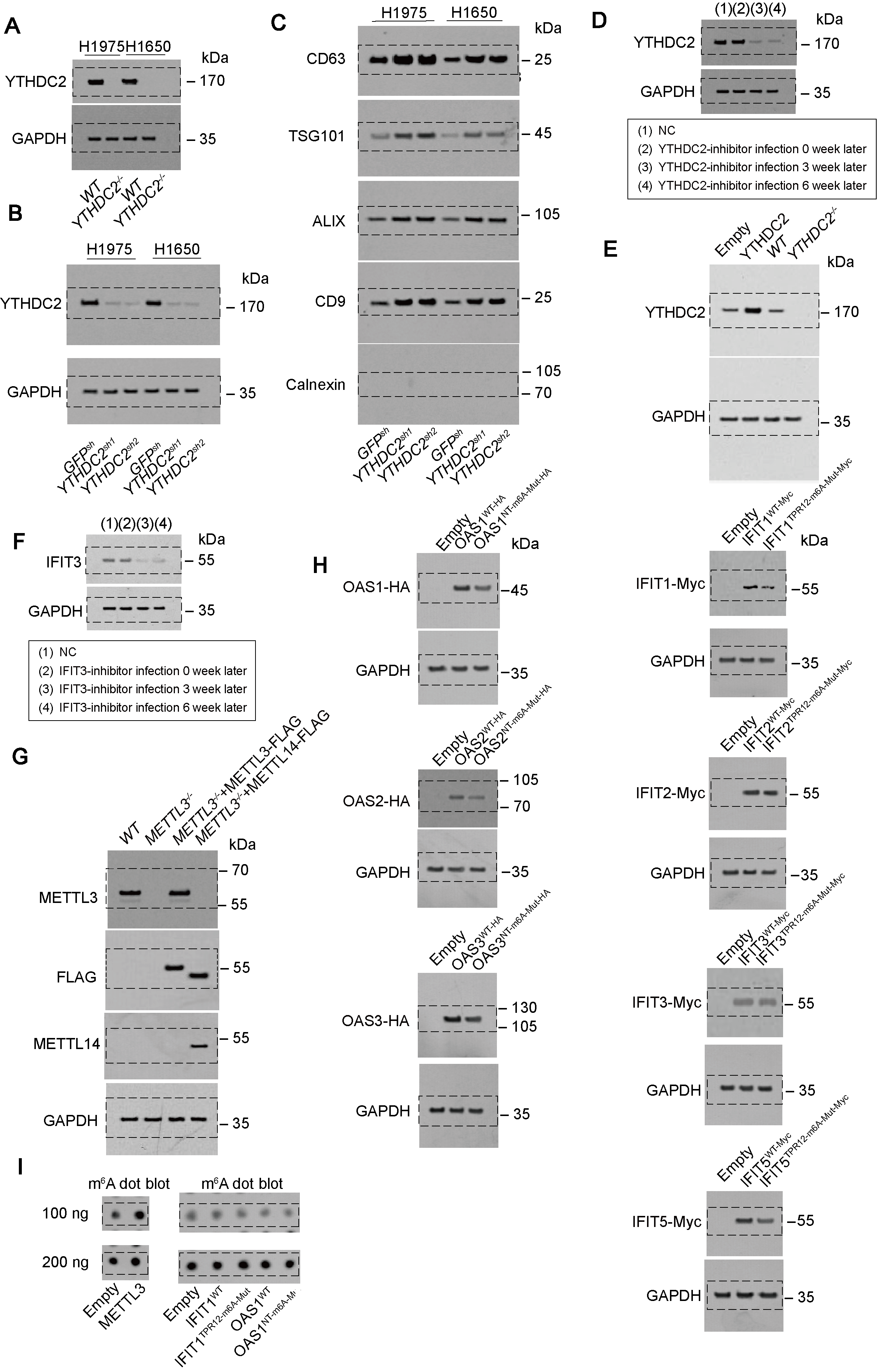
**

**
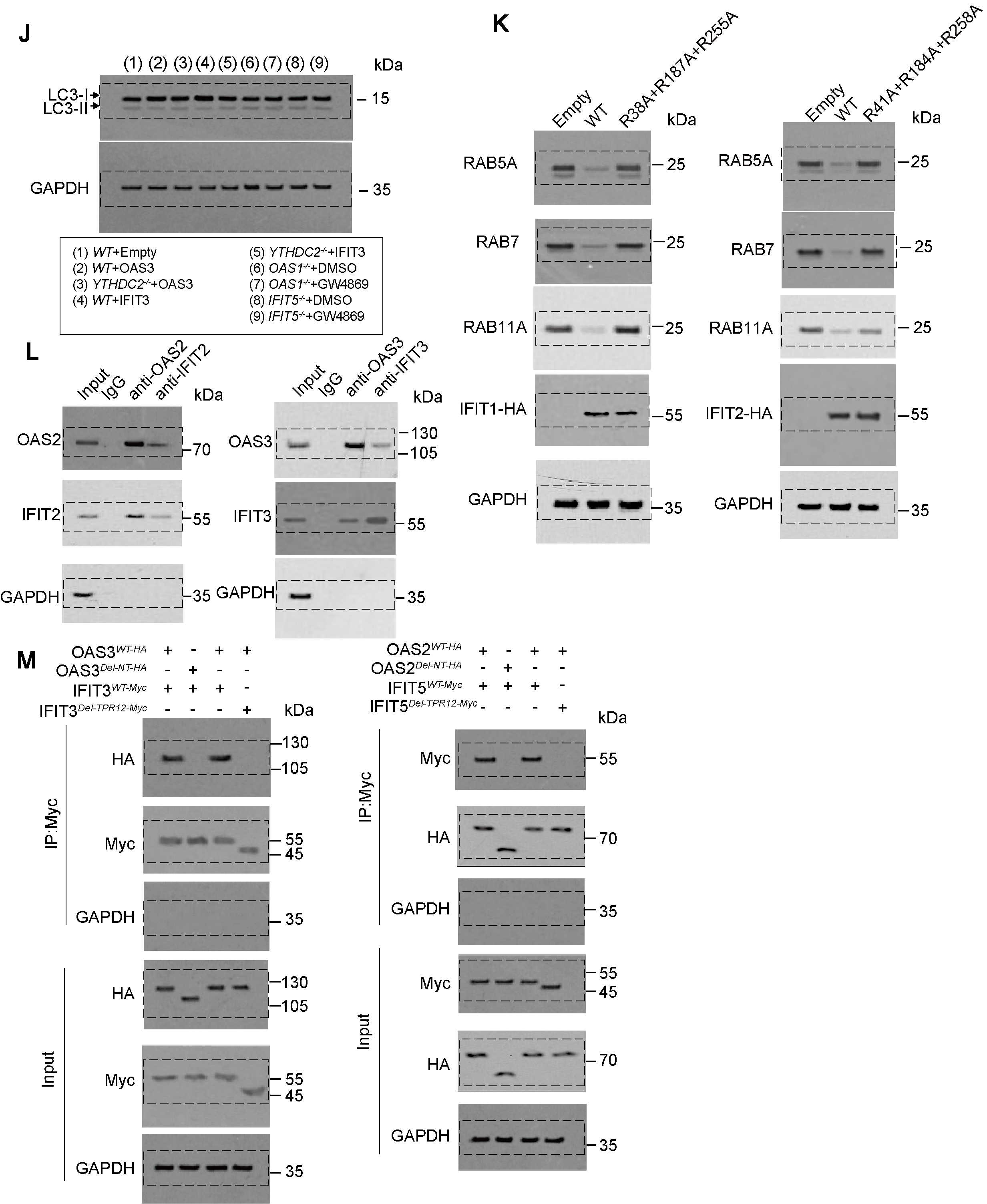
**

**
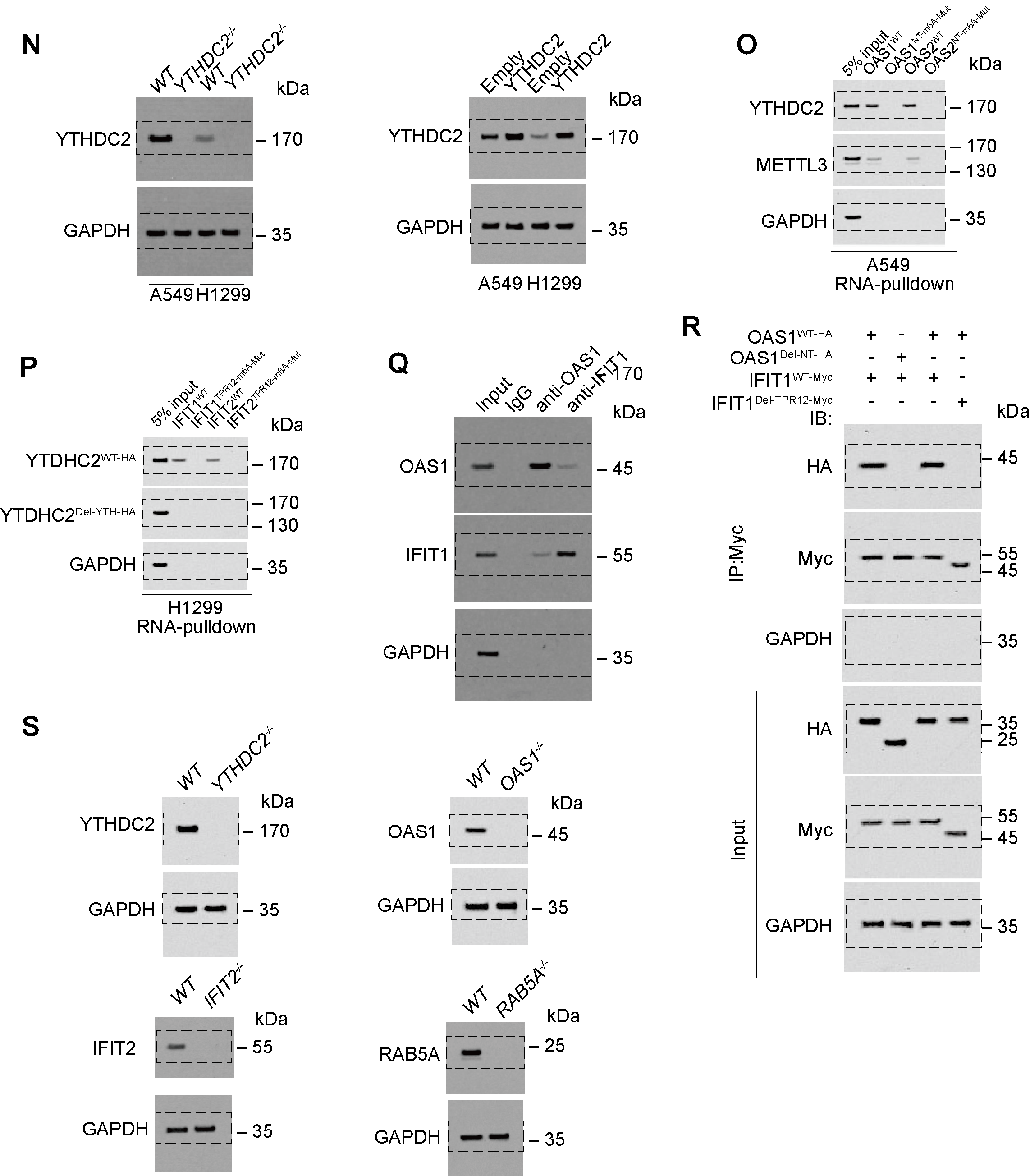
**

**Blots 2: Images for original blots 2**

(A) Original blogs for Fig. S1B.

(B) Original blogs for Fig. S1G.

(C) Original blogs for Fig. S1L.

(D) Original blots for Fig. S1O.

(E) Original blots for Fig. S2C.

(F) Original blots for Fig. S3D.

(G) Original blots for Fig. S4A.

(H) Original blots for Fig. S4D.

(I) Original blots for Fig. S4F.

(J) Original blots for Fig. S6I.

(K) Original blogs for Fig. S7E.

(L) Original blogs for Fig. S8B.

(M) Original blogs for Fig. S8C.

(N) Original blots for Fig. S9A.

(O) Original blots for Fig. S9I.

(P) Original blots for Fig. S9J.

(Q) Original blots for Fig. S9e.

(R) Original blots for Fig. S9f.

(S) Original blots for Fig. S10A.
